# Thermal Kinetics and Nitriding Effect of Ammonia-Based Direct Reduction of Iron Oxides

**DOI:** 10.1021/acssuschemeng.4c02363

**Published:** 2024-06-17

**Authors:** Matic Jovičević-Klug, Yan Ma, Patricia Jovičević-Klug, J. Manoj Prabhakar, Michael Rohwerder, Dierk Raabe

**Affiliations:** † Max Planck Institute for Sustainable Materials (new name), Düsseldorf 40237, Germany; ‡ Max-Planck-Institut für Eisenforschung (old and legally binding name), Düsseldorf 40237, Germany; § Alexander von Humboldt Research Fellow, Alexander von Humboldt Foundation, Jean-Paul-Straße 12, Bonn 53173, Germany

**Keywords:** ammonia-based direct reduction, nitriding, ammonia decomposition, reoxidation, sustainable steel, iron oxide, microstructure

## Abstract

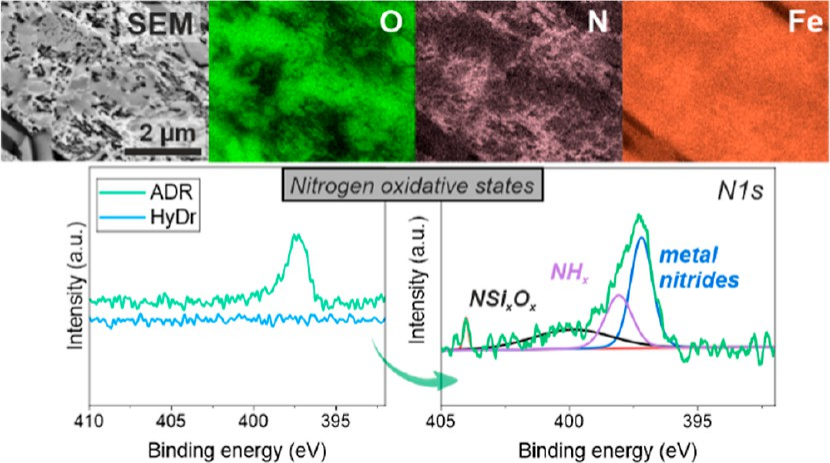

Ammonia is a promising alternative hydrogen carrier that can be utilized for the solid-state reduction of iron oxides for sustainable ironmaking due to its easy transportation and high energy density. The main challenge for its utilization on an industrial scale is to understand the reaction kinetics under different process conditions and the associated nitrogen incorporation in the reduced material that originates from ammonia decomposition. In this work, the effect of temperature on the reduction efficiency and nitride formation is investigated through phase, local chemistry, and gas evolution analysis. The effects of inherent reactions and diffusion on phase formation and chemistry evolution are discussed in relation to the reduction temperature. The work also discusses nitrogen incorporation into the material through both spontaneous and in-process nitriding, which fundamentally affects the structure and chemistry of the reduced material. Finally, the effect of nitrogen incorporation on the reoxidation tendency of the ammonia-based reduced material is investigated and compared with that of the hydrogen-based reduced counterpart. The results provide a fundamental understanding of the reduction and nitriding for iron oxides exposed to ammonia at temperatures from 500 to 800 °C, serving as a basis for exploitation and upscaling of ammonia-based direct reduction for future green steel production.

## Abstract 2

The product of the ammonia-based reduction of iron oxides can be modified by nitriding, bringing new avenues toward protecting porous sponge iron from reoxidation that potentially eases the challenge in storage and transportation of sponge iron.

## Introduction

The global steel industry is facing significant changes to improve its sustainability by cutting the use of fossil carbon-based processes, an unavoidable step to arrive at a lower carbon footprint of this sector.^1,2^ Currently, steel production contributes a whopping 3.73 billion tonnes of CO_2_ per year^3^ due to the high global demand of nearly 2 billion tonnes per year since 2021.^4^ Additionally, the steel market is annually increasing by about 3–4%^4^ translating to a commensurate increase of CO_2_ emissions, if sustainable changes are not implemented. Conventional steel production pathways utilize fossil carbon in the form of coal, coke, and/or methane-based gas mixtures, which is mixed with the iron oxides and heated to a high temperature in either a blast furnace or a shaft furnace. The carbon reacts with the oxide to form CO (Fe_2_O_3_ + 3C → 2Fe + 3CO) and CO_2_ (Fe_2_O_3_ + 3CO → 2Fe + 3CO_2_), resulting in the reduction of iron oxide to an iron-rich product. A large portion of the CO_2_ emissions (about 79%^3,5^) from the steel industry originate from the initial conversion of iron ores (mainly hematite, Fe_2_O_3_) to iron via the blast furnace and basic oxygen furnace route.

As an alternative to this standard production route and means of reducing carbon emissions, hydrogen gas can be utilized in a solid-state direct iron reduction process, named hydrogen direct reduction (HyDR), that is now being implemented around the globe in the steel industry.^6^ The resulting reaction between hydrogen and oxygen from the oxide forms water (Fe_2_O_3_ + 3H_2_ → 2Fe + 3H_2_O), which has no significant environmental impact. Additionally, the resulting water can be utilized for the circular and green production of hydrogen via hydrolysis that can be fed back into the reduction process.^7−9^ However, HyDR comes with many challenges related to both production and storage of hydrogen that will have a significant influence on the successful integration and financial outcome of HyDR, especially with geopolitically set transcontinental green and blue hydrogen production and trade.^10−12^

Ammonia is sought as an alternative hydrogen carrier^13^ for iron oxide reduction. Ammonia can be easily transported due to the existing infrastructure, which originates from the fertilizer and chemical industries. Thus, deploying this medium for ironmaking via ammonia-based direct reduction (ADR) would provide an attractive solution to the decarbonization challenge in the steel industry. However, the production of ammonia via the established Haber–Bosch processes is CO_2_-intensive.^14^ Globally, 2.86 tonnes of CO_2_ are generated when one tonne of ammonia is produced.^15^ This fact means about 0.9 tonnes of CO_2_ emissions without billing in the additional emissions for heating the furnaces when one tonne of steel is produced using ammonia. For comparison, if the ammonia production process were carried out using gray hydrogen, then the emissions originating from the oxide reduction would be reduced by 30%, to around 0.6 tonne of CO_2_ per tonne of steel.^16^ Despite the currently slightly higher CO_2_ emissions compared with methane,^17^ the utilization of ammonia for the current transition of the steel industry from blast furnace operations to hydrogen-based reduction schemes is still massively advantageous. Additionally, in the projected transition period that will most likely take several decades, ammonia can be a viable solution to certain regions of the world and can constitute one of the several pathways that can help to solve the huge challenge of CO_2_ reduction in the steel industry.^6^ Furthermore, new technologies and developments of a more efficient Haber–Bosch process are on the rise and have also become a high interest subject for the scientific community and are supported by local governments in different parts of the world.^14,18−20^ All these points associated with the use of ammonia project this feedstock type as a viable alternative for sustainable iron reduction, with the potential to become established on a global scale. However, it should be clear that ammonia gas usage for direct reduction has other challenges in terms of usage and its potential application compared with hydrogen- and methane-based gas mixtures. A deeper discussion on this topic is provided in Text S1.

The challenges related to technology and reduction capacity follow a similar trend as that of HyDR of iron oxides.^21^ More specifically, the effects of temperature, pressure, and gas concentration on the reactions taking place within a pellet are essential as these will directly dictate the complete process from the design and operation points of view. To deliver insights into the ADR process, this research focuses on two main aspects. The first one is to investigate the fundamental mechanisms that control the reduction capabilities of iron oxides with ammonia at temperatures from 500 to 800 °C. Particular attention will be paid to the microstructural transformations controlled by the chemical interaction of the oxide material with the reducing gas during the reduction process that stems from the ammonia decomposition into hydrogen and nitrogen species in dependency of temperature. The second aspect relates to the final nitriding effect during cooling, which has been documented in previous research.^13^ The effects of such nitriding on the final state of the reduced material and the possibilities of avoiding this final nitriding by gas purging with inert Ar gas are investigated. The implications of nitriding are also explored in relation to the reoxidation properties of the final reduced ore and compared with the reduced material via HyDR.

## Methods

### Material and Reduction Experiments

The reduction was performed in a thermogravimetric analysis (TGA) system comprised of a quartz tube furnace with an infrared heating unit and a weight balance. The experiments were performed on individual commercial hematite pellets with weight ranging from 2.6 to 2.9 g and an average diameter of 11 mm. The chemical composition of the pellets is provided in previous research.^13^ The reduction experiments were performed with a heating rate of 5 °C/s up to temperatures from 500 to 800 °C with a holding time of 1 to 10 h (depending on individual experiments) at a pressure of 1 bar. For the HyDR process, 99.999% pure H_2_ gas was continuously used and for the ADR process, 99.999% pure NH_3_ gas was continuously used during the entire reduction procedure. The reduction gas was introduced to the system, and the chamber was flushed completely before the heating of the samples occurred. The flow rate for the reduction and purging gases was 10 L/h (0.166 SLM). After the holding time, the infrared heating was switched off and the samples were furnace-cooled with the assistance of the room-temperature gas influx (H_2_ for HyDR and NH_3_ for ADR). For individual ADR experiments, purging was also performed with 99.999% Ar gas for 30 min before the cooling procedure, and thus, the cooling to room temperatures was performed subsequently in Ar gas. During the complete reduction process, the weight change of the samples was continuously measured. To minimize the effect of the different gas influx on the sample material and holder in terms of drag and buoyancy changes, the calibration of the system and gas flux adjustment was priorly performed on an empty sample holder and on a pretrial sample pellet. Temperature monitoring and regulation was performed through a thermocouple situated in an additional dummy pellet for tracing the temperature of the interior of the pellet samples. Additionally, for all of the experiments, the gas composition in proximity to the pellet was monitored with a quadrupole gas mass spectrometer with a quartz capillary gas inlet. The measurement of the gas composition was performed in a qualitative manner. The evaluation of the data in relation to the trends in the gas composition changes with the reduction time was performed in a semiquantitative manner by utilizing the ratios between the different gases, which renders the influence of the initial residual gas artifacts as negligible toward the response of the gases to the reduction degree.

### Microstructural Characterization and Local Chemical Probing

The samples were prepared into 1–2 mm-thick disk-shaped samples extracted from the middle of the pellet samples. The sample surfaces were metallographically prepared with grinding and polishing and finalized with silica particle OPS polishing. The metallographic investigation of the samples was performed with a Zeiss Merlin microscope equipped with energy-dispersive X-ray spectroscopy (EDS). The acceleration voltage was in the range of 10–15 kV. For assessment of the average local chemical composition at different portions of the pellets, several measurements on areas of 50 × 50 μm^2^ were performed across the designated regions of the pellets. For confirming proper probing of nitrogen from the different sections of the samples, individual measurements of nitrides specific to the samples [that were determined from X-ray diffraction (XRD)] were used as calibration points to obtain reliable references for assessing the overall nitrogen content from the probed regions of the samples.

For cross-sectional analysis of the reoxidized samples, the samples were wrapped in copper tape and then prepared with standard metallographic preparation. The copper tape acted as a protective layer against local reoxidation and preventive measure against delamination of the reoxidation products during sample preparation. Additionally, copper tape was used to avoid charging of the sample surface during imaging.

### Phase Identification and Quantification Using XRD

The metallographically prepared cross sections of the reduced pellets were directly probed via XRD measurements in a line-scan manner. The XRD was performed with a Rigaku SmartLab 9 kW diffractometer in a parallel beam configuration with a Cu K_α_ source. The 2θ scan range was set from 10 to 100° with a sampling step of 0.01° and scan speed of 2°/min. The line scan across the samples’ surfaces was performed with a slit area of 0.5 × 0.5 mm^2^ and lateral step size of 1 mm. The XRD data was analyzed with Rietveld refinement,^22^ which presented a limit of quantification of 0.2 wt %, with 50% relative error for values up to 0.5 wt %. The average relative error of the XRD quantification from 0.5 to 2 wt % was on average 25%, from 2 to 5 wt %, the average relative error was 10%, and above 5 wt %, the average relative error was 5% or below. For chemical composition assessment of the analyzed cross sections, the nominal stoichiometric compositions of individual phases were used for the calculations. The same data sets were also analyzed for lattice expansion of the iron phase with incorporation of nitrogen. For this purpose, the alignment of the individual diffractograms was performed through centering the spectra to the {211} α-Fe diffraction peak in order to compensate any measurement artifacts between the measured points. The use of this diffraction peak is considered valid for the use as an anchor point for assessing the lattice expansion of α-Fe with nitrogen due to the low shifting of it with nitrogen content^23^ and due to the same, positive, shifting of the peak with increasing nitrogen content as for the {101} peak that was used to assess the lattice expansion through *d*-spacing measurement. From the XRD measurements of the sample fully reduced with ammonia at 700 °C and cooled in Ar gas, the finally reduced iron with low nitrogen content (high purity) was determined as being closest to iron with lattice parameter of *a* = 2.8654 Å (reference 9006587 from COD database) that was used as a reference for assessing the lattice expansion of the iron with nitrogen incorporation.

### Reoxidation Experiments

The reoxidation of the reduced pellets at 700 °C with hydrogen and ammonia gases was performed by immersing the individual samples into a 0.1 mol NaCl water solution for 1 h at room temperature. The utilization of a water solution was primarily used to assess the reoxidation of the reduced material that thus also provides the direct implications on the pyrophoric reaction of the material under wet conditions. For these experiments, a central section of 1 mm thickness was cut out from the individual pellets and ground with grit papers down to 2500 grit. After the immersion, the samples were removed and dried before further analysis and probing was performed. After the removal of the samples from the solution, the samples were stored in a dry environment to minimize the progression of the reoxidation of the samples in an ambient environment.

### Raman Spectroscopy

Raman spectroscopic analyses were conducted using a LabRAM confocal Raman microscope system manufactured by Horiba Jobin Yvon, France. The system was equipped with a 50× objective lens, a 600 L/mm grating, and a charge-coupled device (CCD) detector. The measurements employed a HeNe laser operating at a wavelength of λ = 633 nm. The point incident laser power was approximately 6 mW, focused onto a sample area of 10 μm^2^. The data was collected with an accumulation time of 3 × 600 s and a spectral resolution of around 1 cm^–1^.

The mapping of samples was done by the Confocal Raman microscope system with a green laser by a wavelength of λ = 785 nm, and the power of the laser was 6 mw, focused in the area of 10,000 μm^2^. The objective lens was 50× with 600 L/mm grating and a CCD detector. The data was accumulated during several measurement series with an accumulation time of 3600 s and a spectral resolution of roughly 1 cm^–1^. Raman spectroscopy identification of minerals and spectral fitting was performed based on the specific spectral patterns of the individual mineral type, chemical composition, and polymorphic form.^24^ The identification of these minerals was correlated with the Raman spectroscopic database library RRUFF.^25^

### X-ray Photoelectron Spectroscopy

X-ray photoelectron spectroscopy (XPS) was carried out in a Physical Electronics PHI Quantera II spectrometer equipped with an Al Kα source at 1486.6 eV. For the recording of oxygen (O 1s) and nitrogen (N 1s) core-level spectra, a pass energy of 26 eV was utilized. The iron (Fe 2p_3/2_) core spectra were obtained using a pass energy of 55 eV. The acquisition of the survey scan spectra involved a pass energy of 112 eV. All measurements were conducted with a takeoff angle of 45°. High-resolution core-level spectra for O 1s and C 1s were recorded with an energy step size of 0.025 eV and Fe 2p_3/2_ spectra with an energy step size of 0.05 eV, and survey scan spectra were collected with an energy step size of 0.1 eV. XPS depth profiling was carried out by etching the sample with an Ar ion beam of energy 2 kV on an area size of 2 mm × 2 mm for different time intervals (0–360 s).

The peak fitting of the core spectra from XPS studies was performed using CasaXPS software version 2.3.22, with a Shirley-type background applied to all analyzed spectra. Symmetric components utilized a Gaussian (70%)—Lorentzian (30%) peak shape, and the metallic iron component was fitted using a Lorentzian Asymmetric, LA(1.3, 4, 5), peak shape to accommodate the asymmetric metallic peak.

## Results and Discussion

### Temperature-Dependent Reduction Performance and Relation to Gas Evolution

**Figure 1:**
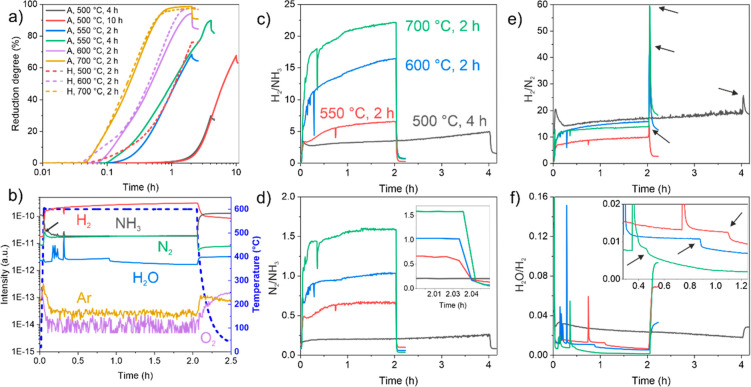
(a) Reduction kinetics of hematite pellets reduced with hydrogen gas (H), marked with dashed curves, and ammonia gas (A), marked with solid curves, at different temperatures. (b) Gas evolution during ADR of the hematite pellet at 600 °C for 2 h, probed by in situ mass spectrometry. (c–f) Evolution of gas intensity ratios of (c) N_2_/NH_3_, (d) H_2_/NH_3_, (e) H_2_/N_2_, and (f) H_2_O/H_2_ for ADR samples reduced at 500, 550, 600, and 700 °C. The arrows in (b,e,f) indicate the positions of the discontinuities of the ratios of traced gas signals. The annotations from the curves in (c) are the same for the other color-coded curves in (d–f). In all cases, the ADR samples were cooled with ammonia gas.

The reduction experiments of iron ore pellets with HyDR and ADR from 500 to 700 °C, presented in Figure 1a, reveal that at higher temperatures (600 and 700 °C), the reduction kinetics are similar regardless of the reduction media used (namely, hydrogen and ammonia). However, at the lower temperatures of 500 °C, the reduction kinetics are much slower for the ADR (red solid curve in Figure 1a) than for the HyDR case (the red dashed curve). For comparison, the HyDR kinetics at 500 °C are similar to the kinetics of ADR at 550 °C (Figure 1a), clearly indicating the faster weight loss with HyDR compared with ADR at lower temperatures. The differences between HyDR and ADR are particularly visible in the initial stages of the reduction, which is seen by the stronger temporal offset in the initiation of the reduction of the pellet for ADR compared to HyDR. As seen from Figure 1a, the offset is primarily visible with lower temperatures of 600 °C and below, whereas the reduction curves are similar for the 700 °C cases. The origin of the offsets is related to the limited ammonia decomposition as well as to the simultaneous nitriding, both of which become increasingly more prominent with lower reduction temperatures. The effect of the individual processes on the reduction degree is further described in Text S1. However, it is worth noting that the reduction degree is calculated from the weight change of the sample, which does not necessarily constitute a direct relation with the mass loss from reduction (i.e., oxygen removal), particularly when simultaneous nitriding occurs at lower temperatures in ADR, i.e., weight gain, because of nitrogen incorporation.

The effect of nitridation with ADR is further visible at the end of the reduction process. A clear sudden drop in the reduction degree (related to an increase in the weight of the sample) during cooling becomes visible for the ADR samples, as seen in Figure 1a. Based on previous observations of ADR conducted at 700 °C,^13^ this phenomenon has been associated with the spontaneous formation of nitrides during cooling in ammonia. The experiments at lower temperatures reveal that the final nitridation effect is strongly dependent on the reduction temperature as well as on the reduction degree of the sample after which cooling takes place (see solid curves in Figure 1a and extracted data presented in Table 1). The extracted values of the reduction degree change presented in Table 1 clearly show that with a higher reduction degree, the final nitriding effect becomes proportionally larger also. However, a discrepancy is visible for the sample reduced at 600 °C at 2 h that showed the highest change due to the spontaneous nitriding. To evaluate this trend further, experiments were carried out at 800 °C (not shown in Figure 1a) to provide evidence that the spontaneous nitriding does, in fact, decline at temperatures above 600 °C, as can be seen in Table 1. This unique behavior indicates a more complex relation of the spontaneous nitriding effect, which could be related to either the spontaneous gas absorption or changes in the volume of the pellet in terms of increasing the areal resistance to the flowing gas that results in an artificial lowering of the weight of the sample.

**Table 1:** Extracted Values of the Reduction Degree Just before Cooling and after Cooling to Room Temperature of the Samples from Individual Reduction Temperatures and Reduction Times^a^

reduction temperature (°C)	reduction time (h)	maximum obtained reduction degree (%)	reduction degree after cooling (%)	reduction degree change from spontaneous nitriding (%)
500	4	30.0	27.0	–3
500	10	68.4	64.3	–4.1
550	2	67.6	63.0	–4.6
550	4	89.8	82.2	–7.6
600	2	94.0	84.5	–9.5
700	2	98.7	90.7	–8.0
800	1	97.3	91.3	–6.0

a The final column displays the difference between the previously described values, indicating weight gain during the cooling process. The values were extracted from TGA measurements presented in Figure 1a.

Further elucidation of the simultaneous nitriding during reduction and the spontaneous nitriding during cooling is provided by the in situ compositional analysis of the reaction gas in the vicinity of the treated samples (see Figures 1 and S1). As given by the example in Figure 1b, a major change in gas composition occurs during the heating procedure as the temperature reaches temperatures above ∼300 °C, from which the decomposition of ammonia to hydrogen and nitrogen gas starts. However, the yield of decomposition is not a step function, but it is formed with a characteristic rate that is determined by the temperature and interacting material.^26,27^ In these experiments, both iron oxide and the resulting iron can act as catalytic media^26−30^ for the ammonia decomposition because both substances lower the cracking temperature for NH_3_ to around 300–350 °C.^31^ The increasing decomposition of ammonia with temperature can be seen through the ratio of H_2_/NH_3_ and N_2_/NH_3_ gas intensities as presented in Figure 1c,d, respectively. These graphs also indicate that the decomposition is not a continuous process during the reduction at a specific temperature but rather shows different steps during the process.

In the initial phase, when close to the targeted reduction temperature for the cases with temperatures of 550 °C and above, a peak develops in the nitrogen signal (marked with an arrow in Figure 1a), whereas the hydrogen gas signal does not show this feature, as seen from Figure 1b. However, when the reduction is performed at 500 °C, the nitrogen peak does not occur, but instead, a strong peak in the hydrogen signal is observed (Figure S1a,b). The peaking effect is more visible when plotting the ratio of H_2_/N_2_ intensities as given in Figure 1e (see the dark curve). The origin of such gas behavior is considered to be associated with the initial adsorption of ammonia on the oxide surface and dehydrogenization that is modified with the thermal energy of the oxide surface. This is further discussed in the theoretical section of the paper in Text S2.

A particular feature is also that with the reduction progression, a clear steady increase in the hydrogen and nitrogen gas signals is visible up until the end of the reduction process for all temperature cases, as seen from Figure 1c,d. This phenomenon could originate from the slight increase in the ammonia decomposition with the increasing formation of metallic iron surfaces that are considered to have better catalytic efficiency for NH_3_ splitting than iron oxides.^26,29,30^ However, the increase of both gases is disproportionate, with the hydrogen concentration increasing more rapidly than the nitrogen concentration with reduction time, as can be clearly seen from the steady increase in H_2_/N_2_ in Figure 1a for all cases. This phenomenon can be ascribed to the gradual hydrogen gas accumulation within the analysis chamber and capillary system of the mass spectrometer. This is confirmed by the systematically repeating discrepancies seen in the residual hydrogen signal before and after the reduction process, while the other gases such as NH_3_ and N_2_ returned to the same values (see Figures 1b and S1).

The reduction evolution and relation with the interaction of the ammonia and its decomposition products can be seen through the water signal, which can provide clear insights into the kinetics and specific features of the reduction process (see Figure 1b,f). A deep discussion of the water signal correlation to various phenomena is provided in Text S3. The water signal could provide a very suitable method to monitor the reduction degree on an industrial scale, particularly when partial reduction of the pellets would be considered for transportation purposes, which can be further processed with hydrogen-plasma smelting reduction.^32^

The evolution of the gases during the final cooling step provides insights into the possible nitriding during cooling. Figure 1b–d shows the immediate decline of the hydrogen and nitrogen gas signals that follow the cooling of the samples and the reduced efficiency of the ammonia decomposition. However, as shown in Figure 1e, a stronger depletion of nitrogen gas compared with hydrogen gas occurs, which appears as a spike in the H_2_/N_2_ ratio. This could possibly be related to two phenomena. One could be due to the nitrogen uptake by the reduced material that is propelled by the higher nitriding potential originating from the higher *P*(N_2_ + NH_3_)/*P*(H_2_) ratio. However, as can be seen by the inset in Figure 1d, the nitrogen gas is removed very rapidly, reaching 5 times lower signal intensities within a 20 s time span, and it is followed by a similar trend of the hydrogen gas, an effect that can be attributed to the immediate decline in the ammonia decomposition. The second possibility is that this is a measurement artifact resulting from the slower removal of hydrogen gas in the mass spectrometer’s analytical chamber compared with the other heavier gases. This can be seen by the dissimilar signal level of the hydrogen gas before and after the reduction process. In comparison, other gases (nitrogen, oxygen, and ammonia) return to a similar value after the cooling of the sample to room temperature (see Figures 1b and S1), giving a strong support to the previous claim on the measurement artifact. The consideration of the final nitridation is further discussed in Text S2.

### Phase Transitions, Nitriding, and Local Chemical Partitioning at 500 °C

**Figure 2:**
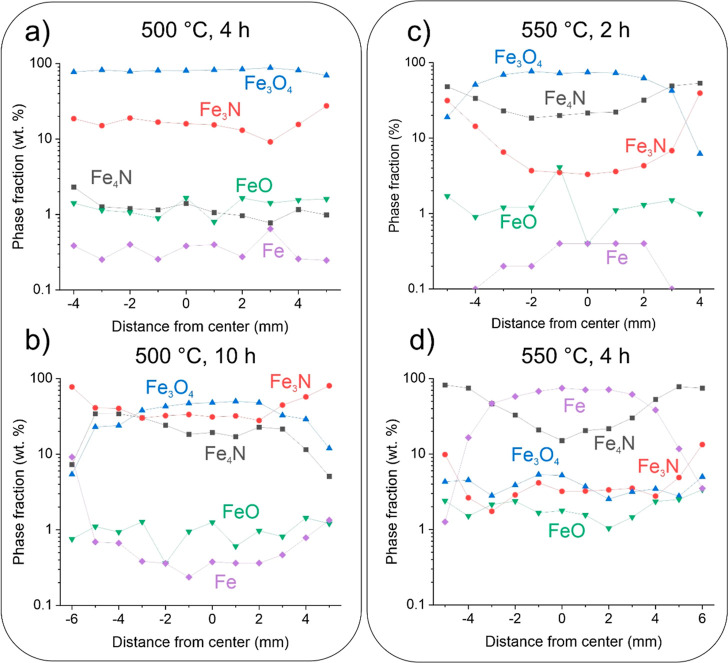
Cross-sectional phase composition of the ADR pellet reduced at (a) 500 °C for 4 h, (b) 500 °C for 10 h, (c) 550 °C for 2 h, and (d) 550 °C for 4 h. The data was extracted from XRD measurements performed in a line-scan manner.

Local XRD analysis performed in a line-scan manner through the cross section of individual pellets clearly presents the shell–core reduction and nitriding behavior. For the samples treated at lower temperatures of 500 °C, the XRD results (see Figure 2a,b) display the distinct formation of Fe_3_N nitrides, originating from the simultaneous nitriding of the oxide material with the ammonia gas and its dehydrogenation and formation of the decomposition products.^31,33,34^ The evolution with time (compare Figure 2b with 2a) indicates an interesting relation to the formation of the nitrides. More specifically, Fe_3_N forms in a trend from the outer surface toward the center, while the Fe_4_N nitrides, that had previously not been majorly detected after ADR at 500 °C for 4 h, form preferentially from the pellet center to the surface. This inverse relationship in the fraction of formation of Fe_3_N and Fe_4_N presents a clear dependence of the spontaneous nitriding on the nitriding potential and the different mechanisms of reduction of the iron oxides related to the nitriding process. The nitriding effect in the basic Fe_3_N structure is heterogeneous both spatially, in terms of its occurrence in the pellet volume, and chemically, in terms of the nitrogen fraction within its primary crystal structure. Individual XRD data (see Figure S3) reveal that the Fe_3_N structures closer to the surface of the pellet present a stronger shift toward larger ordering parameters that constitutes an increased quantity of nitrogen in the form of interstitial solutes in the form of Fe_3_N_1.1–1.3_. This observation indicates that in addition to the nitriding of the pellet material with ongoing NH_3_ and N_2_ gas exposure, also a diffusion-governed migration of nitrogen occurs due to the higher nitriding potential of the surface of the pellet compared with the pellet core.

**Figure 3:**
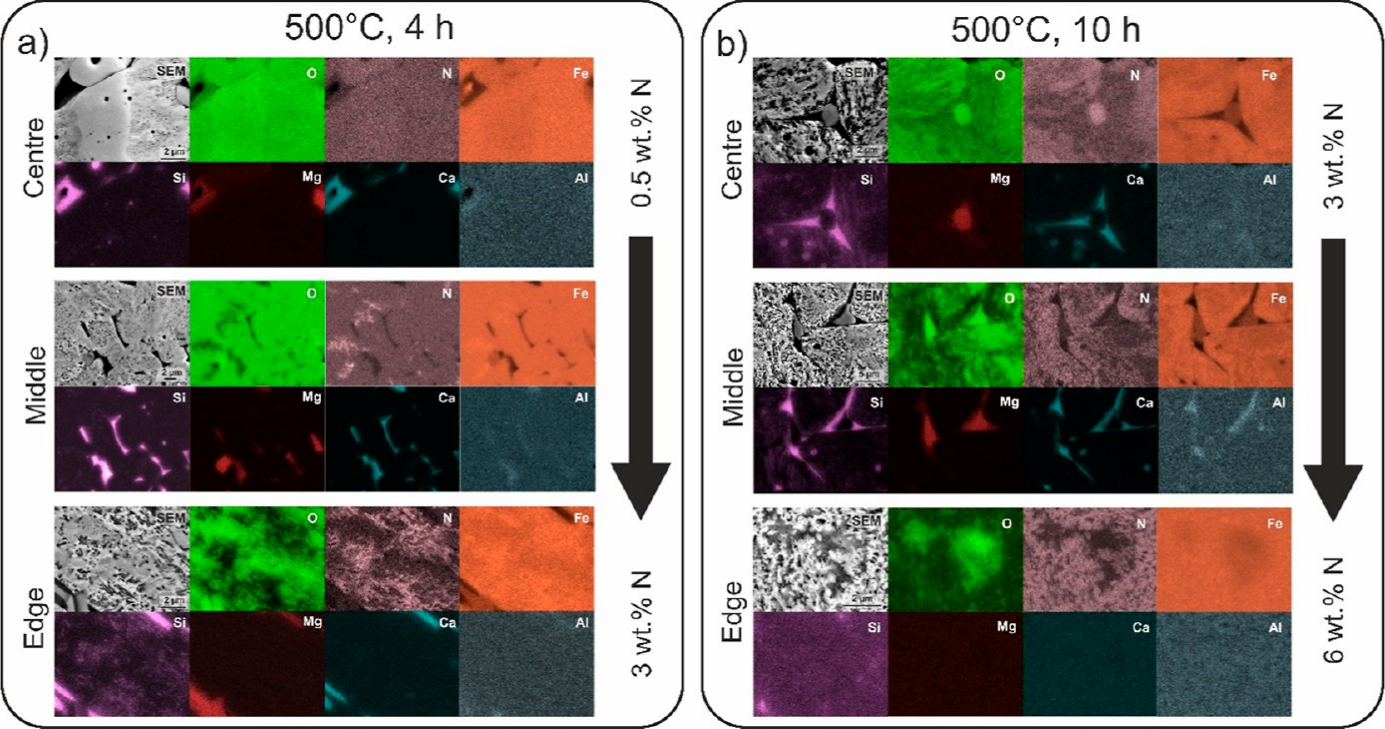
SEI micrographs and elemental distribution maps obtained with EDS for the ADR pellet reduced at 500 °C for (a) 4 and (b) 10 h. The different regions represent a typical microstructure present in the different portions of the pellet. The accompanying maximum and minimum values of nitrogen situated next to the arrow are acquired using quantitative local EDS analysis in the specified different regions of the pellets.

Further evidence is provided by local SEM and EDS analyses which clearly reveals that nitriding occurs within the pellet in a gradient manner. The contrast from secondary electron imaging (SEI) clearly portrays brighter regions that correspond to the nitrides. The nitrides preferentially form as branched structures that expand from pores and voids that are inherently present in the pellet^35^ or formed with progressive reduction of the surrounding oxide.^13,35^ The EDS maps, presented in Figure 3, confirm this relation and show that the intensity of the nitrogen signal is less pronounced deeper inside the pellet despite the presence of the nitride structures, visible through SEI. This feature coupled with an overall similar nitriding fraction determined with XRD indicates that with deeper penetration into the pellet, the nitrides develop a finer structure (average thickness of 66 ± 15 nm) compared with the outer portions of the pellet (average thickness of 176 ± 47 nm). This pairs together with the more inhomogeneous formation of nitrides in the inner portion of the pellet that is also shown by SEM probing and quantitative nitrogen content analysis (Figure 3). The effect of the diffusional nitriding of the pellet material and subsequent formation of Fe_4_N is visualized with SEI and EDS of the reduced pellet at 500 °C for 10 h (Figure 3b). See the enlarged micrograph with marked nitrides in Figure S4 for more details. SEI clearly shows that the branched structures, associated with the Fe_3_N, form preferentially in the outer portions of the pellet and are accompanied by a periphery of Fe_4_N, which develops as a transition structure between the Fe_3_N and the remaining magnetite (Fe_3_O_4_) patches. Such a relation indicates that there is an inherent exchange of oxygen with nitrogen that occurs in a diffusional manner and is controlled by the temperature as well as by the excessive amount of nitrogen in the surrounding material. The nitrogen gradient is further confirmed through EDS probing of larger areas of the reduced sample sections as schematically depicted in Figure 3a,b with the large arrow. Such analysis also confirms that the nitrogen content increases with a longer reduction time that coincides with the higher nitride fraction in the different parts of the samples.

### Phase Transitions, Nitriding, and Local Chemical Partitioning at 550 and 600 °C

The temperature dependence of the nitriding process is further evidenced by the examination of samples reduced at 550 °C for 2 and 4 h. The XRD results (see Figure 2c,d) display a profoundly different nitride evolution compared to the samples that were reduced at 500 °C. As Figure 2c shows, the residual magnetite composition of the sample reduced at 550 °C for 2 h is very similar to the 500 °C 10 h sample, which coincides with a similar reduction degree determined from the TGA measurements (see Figure 1a). However, the fraction of Fe_3_N is much lower and the Fe_4_N profile for the 550 °C sample presents an enrichment closer to the surface, which is inversely related to the Fe_4_N profile from the samples reduced at 500 °C for 10 h (see Figure 2b). Additionally, the Fe_4_N fraction is overall higher than that of the Fe_3_N fraction observed in the 550 °C sample. These results indicate that at 550 °C, the Fe_3_N nitride is thermodynamically unstable and that the Fe_4_N phase forms preferentially over the Fe_3_N, related to the nitriding potential as described by the Lehrer diagram.^36^ This is further evidenced by the reduced volume fraction of Fe_3_N with progression of the reduction to 4 h, as presented in Figure 2d. Such behavior follows closely the thermal decomposition of Fe_3_N species as determined with in situ neutron diffraction conducted by Widenmeyer et al.^37^ XRD analysis also reveals that the reduction has mostly finished, as seen by the few wt % of remaining oxides (Fe_3_O_4_ and FeO) throughout the sample cross section, translating to a low remaining oxygen content as also seen from the stoichiometric calculation presented in Figure S5. The XRD mapping also discloses that after 4 h of reduction, α-iron (ferrite, bcc structure) is formed and remains stable within the sample despite the high nitriding potential, thus supporting the destabilization of the Fe_3_N with progressive reduction at 550 °C. In addition, such information indicates that the spontaneous nitriding event does not occur in a homogeneous fashion and that rather the nitriding is an effect of the diffusional-guided alloying of the iron with nitrogen over time. Local EDS mapping (provided in Figure S6) also clearly displays the gradient in the nitrogen signal, increasing from the center to the outer rim of the reduced pellet. The localized EDS measurement of the individual regions of the pellet confirms that the nitrogen content is also considerably higher, reaching values up to 6 wt % at the edge of the pellet. Additionally, the central portion displays on average about 1 wt % of nitrogen, which correlates well with the determined phase fractions of Fe_4_N and Fe from XRD (see Figure 2d).

**Figure 4:**
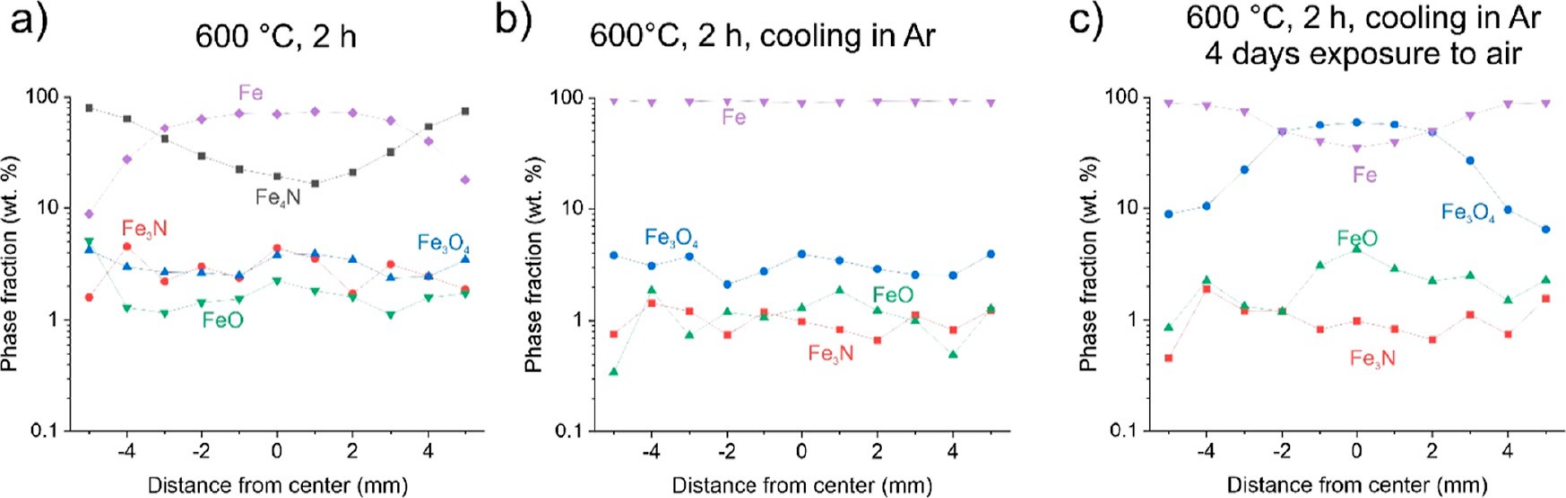
Cross-sectional phase composition of ADR pellet samples reduced at (a) 600 °C for 2 h and (b) 600 °C for 2 h with cooling performed in Ar gas. The sample from (b) was exposed for 4 days to ambient conditions (room temperature and average humidity of 60%) and the cross-sectional phase composition was re-evaluated. The data was extracted from XRD measurements performed in a line-scan manner.

**Figure 5:**
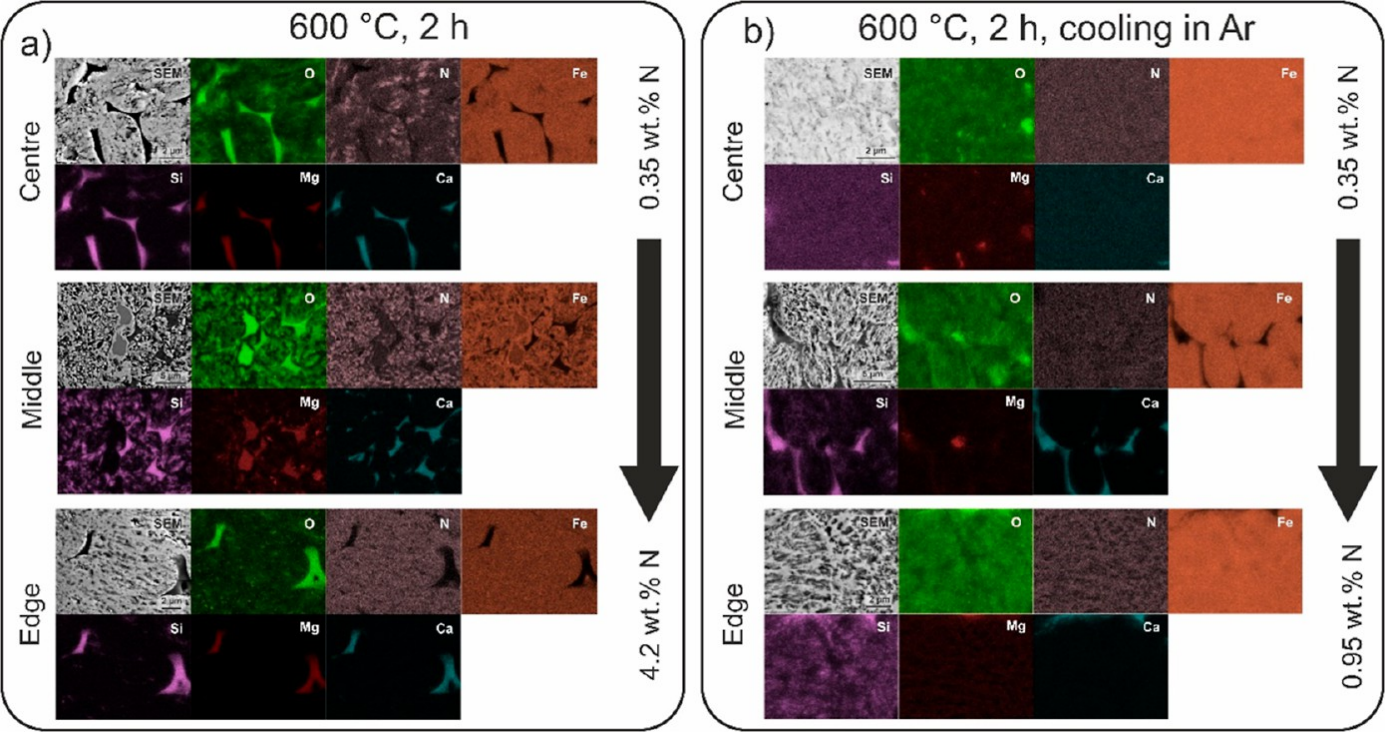
SEI micrographs and elemental distribution maps obtained with EDX for the ADR pellet reduced at 600 °C for 2 h by cooling in (a) NH_3_ gas and (b) Ar gas. The different regions represent a typical microstructure present in the different portions of the pellet. The accompanying maximum and minimum values of nitrogen situated next to the arrow are acquired with local EDX quantitative analysis in the differently specified regions of the pellet.

**Figure 6:**
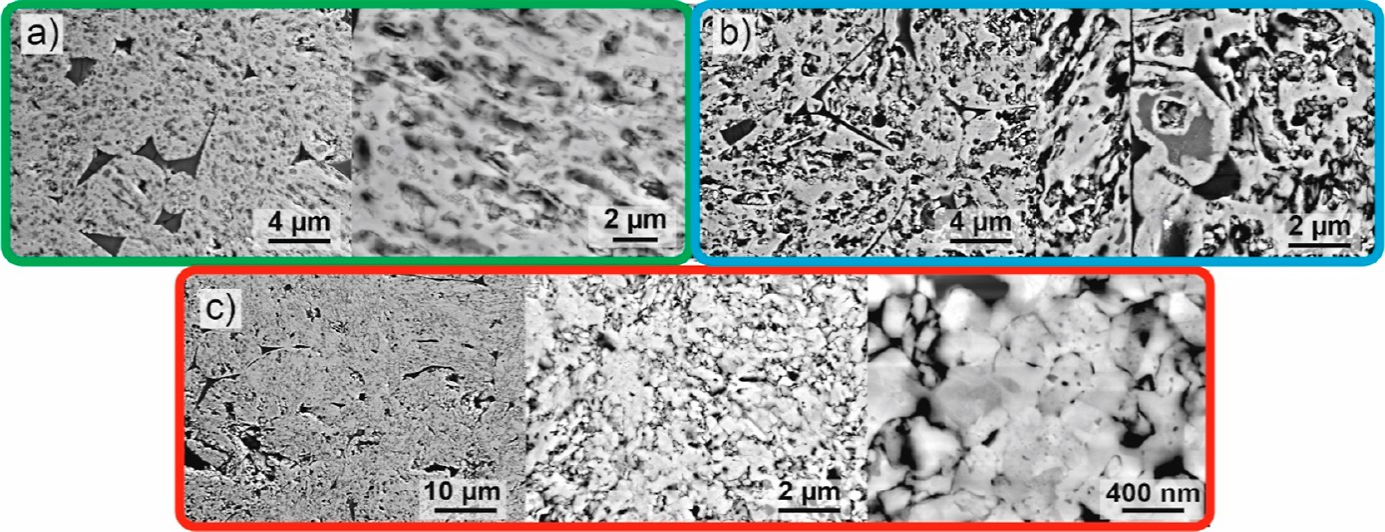
Representative SEI of the different sections: (a) edge, (b) middle, and (c) center of the ADR pellet reduced at 600 °C for 2 h.

With the increase of the reduction temperature to 600 °C, the Fe_3_N phase is practically no longer present after 2 h of reduction, due to the decreasing thermodynamic stability of this nitride type with higher temperature.^37^ In exchange, the Fe_4_N is the only nitride considered to be present in the microstructure in significant quantities next to the iron as seen from Figure 4a, which as discussed later forms due to the cooling of the pellet in a nitriding atmosphere. Interestingly, the 2 h reduction at 600 °C results in a similar cross-sectional phase composition with respect to iron and Fe_4_N as that of the 550 °C 4 h reduced sample (see Figure 2d). Similarly, the overall extracted chemistry from the cross section is nearly identical (Figure S5), despite the slight differences in the TGA results in terms of the reduction degree. This comparison clearly shows the strong impact of the temperature on the reduction kinetics, which also influences the morphology of the phases and reduced material, as visible from Figure 5a. The main difference lies in the much higher degree of condensed material forming in the center of the pellet because of localized diffusional exchange of iron, nitrogen, and oxygen. The exemplar high-resolution SEM images of the reduced pellet in Figure 6 clearly depict the strongly modified morphology and grain size effects in different sections of the reduced pellet. In Figure 6a, the stark porosity formation at the pellet periphery in the form of regular rounded shapes is visible. Such morphology originates from the instantaneous nitriding and reduction that can form through intermediate steps, such as the decomposition from Fe_3_N to Fe_4_N, due to the direct interaction with ammonia gas. With transition toward deeper parts of the reduced pellet, the porosities are on average larger and more interconnected compared to the immediate edge of the pellets, as seen from Figure 6b. The origin of the different porosity yield in this part is related to the change of the nitriding through a combination of gas decomposition and nitrogen diffusion from the edge of the pellet.

The latter is clearly registered by the gradient distribution of nitrogen that is seen from the EDS mapping in Figure 5a. Additionally, the entrapment of water- and ammonia-derived decomposition gases can occur in deeper parts of the material, resulting in a complex ensemble of entrapped reducing and nitriding gases that together allow the formation of larger separated domains of nitrides and iron as observed in Figures 5a and 6b. This in turn leads to the buildup of a higher degree of incoherency and the associated elastoplastic mismatch and decohesion of the adjacent phases. Due to the layer-like formation of the individual phases, this effect can result in a higher interconnectivity of the pores and decohesion zones within the material. In addition, the previously discussed core–shell structure of the initial pellet with a higher porosity in the intermediate zone, originating from the sintering process, has additional influence on the formation of this characteristic type of multiphase and free volume morphology. However, it should be clear that the morphology extends beyond the immediate position of such prior structuring of the pellet toward both the edge and center of the pellet. Toward the center of the pellet, the density of the material increases with much more isolated pores and an increased polygonization effect (characterized by a certain decohesion and free volume pattern surrounding the grains and grain clusters), Figure 6. The increased density is postulated to occur due to iron and oxygen diffusional counterflow and local sintering that together compensate the chemical gradients formed in the sample due to the progressing reduction. The nitrogen content in the center of the reduced pellets (0.35 wt %) is considerably lower compared with the outer region of it (4.2 wt %) as seen from Figure 5a, correlating well with XRD results from Figure 4a. However, the nitrogen mostly is related to individually formed nitrides that can be clearly seen from the localized enrichment of nitrogen from the EDS mapping (see Figure 5a). This is most probably related to a strong interconnectivity of the porosity reaching deep into the pellet center that allows direct gas nitriding also in the more central pellet regions.^35,38,39^

### Effect of Cooling Gas and Implication on Reoxidation

To investigate the impact of the cooling on the formation of the nitrides and the residual presence of nitrogen in the reduced hematite pellet, additional experiments with the same reduction times and temperatures were performed with additional purging with Ar for 30 min before the reduced material was cooled to room temperature. The TGA results clearly show that the sudden drop in the reduction degree (mass gain) does not occur as seen from Figure S7a. The effect of Ar purging also shows the steep change in the gas composition (see Figure S7b), an effect that indicates that either the hydrogen is quickly consumed or the decomposition of the ammonia halts due to the lower concentration at the surface of the pellet. Surprisingly, the nitrogen gas displays a different trend compared with the hydrogen gas (Figure S7b), which could indicate a nitrogen degassing effect from the reduced material. Additional support to degassing is seen with the cooling in Ar gas that presents an increase in the NH_3_ signal, which most probably originates from the contraction of the material with cooling that physically pushes entrapped gases from the pores of the reduced pellet. This corroborates with the increased mass loss of the purged sample (see Figure S7a) that is also registered by a positive derivate of mass change compared with the normal negative derivate of mass change seen for the samples cooled in ammonia (compare Figure S7c with Figure S1). This follows well previous results of Iwamoto et al.,^40^ which also presented that prior Ar purging retards nitride formation. The localized XRD results in Figures 4b and S8 also confirm the major presence of only Fe with nearly no nitrides detected for the 600 and 700 °C ADR samples. However, localized EDS results do provide information that despite the lack of nitride phases, the material still holds some amount of nitrogen with slight gradient enrichment from the edge to the center of the pellet (see Figure 5b). To elucidate the origin of the nitrogen presence, the chemical shifting of the main {101} iron peak was evaluated across the cross section of the 600 °C sample reduced for 2 h with cooling in Ar gas. The iron peak displays a clear shift toward lower 2θ angles, caused by a lattice expansion from the center toward the edge of the pellets (see Figure S9). The maximum shift at the edges of the samples corresponds to a lattice expansion of around 0.0019 Å. This translates to nitrogen values of about 1–5 at. % in the ferrite matrix based on the literature,^23^ which falls in the range of 0.2–1 wt % of nitrogen, correlating well with the EDS results of the sample with Ar cooling (see Figure 5b). This finding clearly confirms that not only nitriding occurs as a chemical process and phase transformation effect but nitrogen enters the iron matrix also through diffusional incorporation as a solid solution interstitial that can even supersaturate under conditions with low nitriding potential. The effect of nitrogen incorporation is also detected with exposure of the material to oxidative environments. The prepared cross sections of individual samples were exposed to ambient environments (20 °C in air with an average humidity of 60%) that resulted in a gradient development of magnetite. The gradient was clearly visible through cross-sectional XRD probing (see Figure 4c), which indicates that the iron in the central portion of the pellet oxidized strongly (nearly 50 wt %), while the outer regions were less oxidized (only about 10 wt %). The stronger oxidation of the central portion against the edge region of the pellets was also visualized by local SEM and EDS analysis, as presented in Figure S10. These results also indicate that the oxidation occurs in a patch-like form that correlates with the varying effect of the nitrogen incorporation into the material as seen from the reduced sample material.

### Reoxidation Protection of ADR Pellets through Nitrogen Incorporation

**Figure 7:**
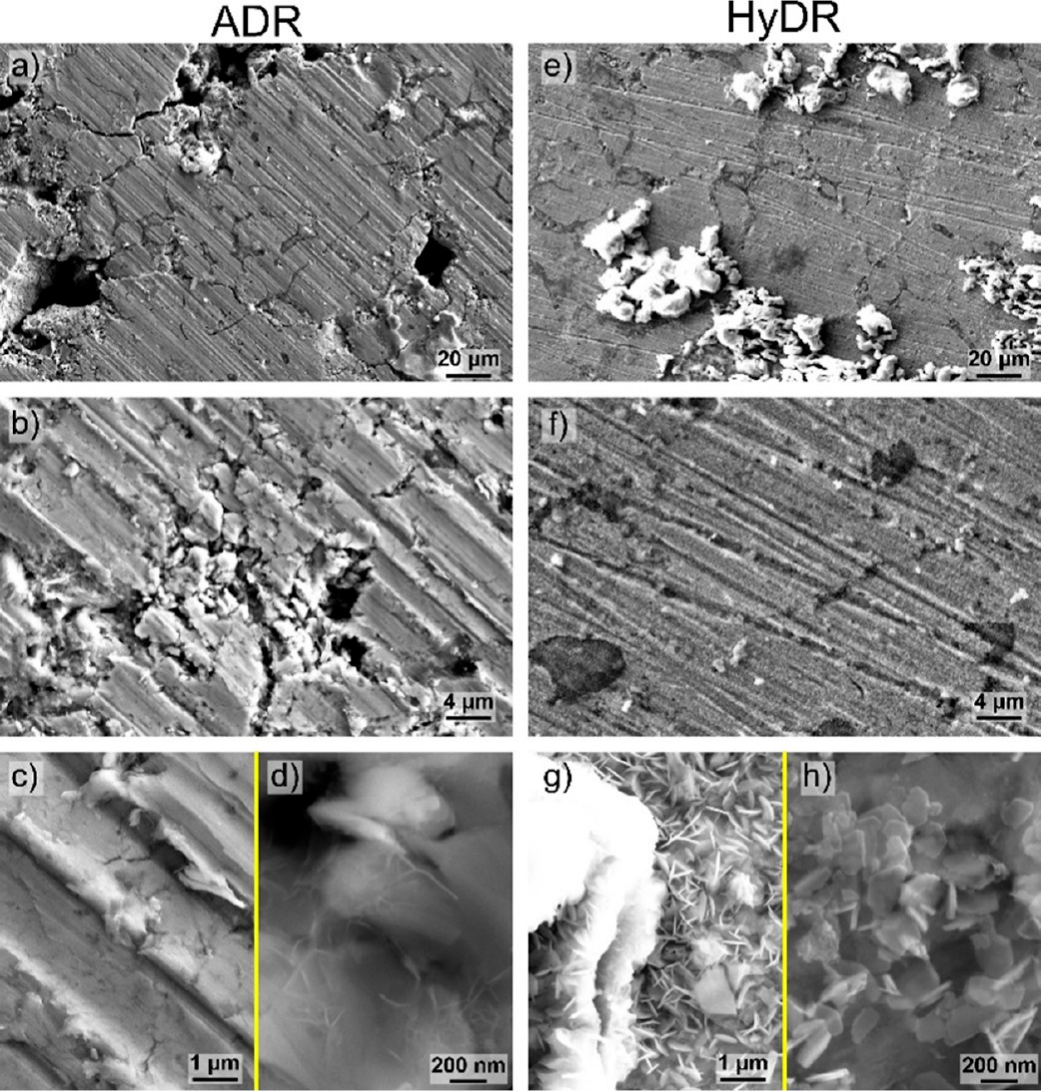
SEM images of reoxidized surfaces of (a–d) ADR and (e–h) HyDR samples.

To test the effects of solute nitrogen incorporation and nitride formation during ADR regarding their possible roles in the protection of as-reduced pellets against reoxidation, fully reduced ADR and HyDR samples at 700 °C were exposed to a NaCl solution (see further details in the Methods section). The resulting buildup of oxides was then characterized to understand the different reoxidation tendencies and to estimate the reoxidation progression for each type of sample. The SEM investigation of the surface of the samples reveals a staggering difference between the HyDR and ADR samples (Figure 7). The ADR sample reveals only minor reoxidation of the top surface as seen from Figure 7a. The topography related to the grinding is mostly conserved and displays small amounts of delamination and flaking (see Figure 7b), which morphologically could be related to the formation of wüstite (FeO).^41,42^ Individual regions displayed excessive oxide buildup that was mostly situated around larger porosities and cracks (see Figure 7a). The smaller cavities also display a small amount of other oxides that morphologically correspond to the magnetite and maghemite phases.^43−45^ In contrast, the HyDR sample developed a thick and mostly uniform layer (see Figure 7e,f) of tetrapodic and plate-like shapes (see Figure 7g,h) that morphologically coincide with magnetite (Fe_3_O_4_).^43,44^

In contrast, the oxide buildup in the case of the HyDR sample is much more dominant, with the surface displaying stronger remodeling that is also visualized by the less pronounced grinding features as seen from Figure 7e. Additionally, the regions with larger pores are majorly displaying even higher degrees of oxide buildup as compared with the ADR sample, displaying oxide buildup with thicknesses exceeding several 10s of μm. As seen from Figure 7e,g, the pores are filled with thick structures that develop typical morphologies and features that coincide with lepidocrocite, akaganeite, and hematite.^44−46^

**Figure 8:**
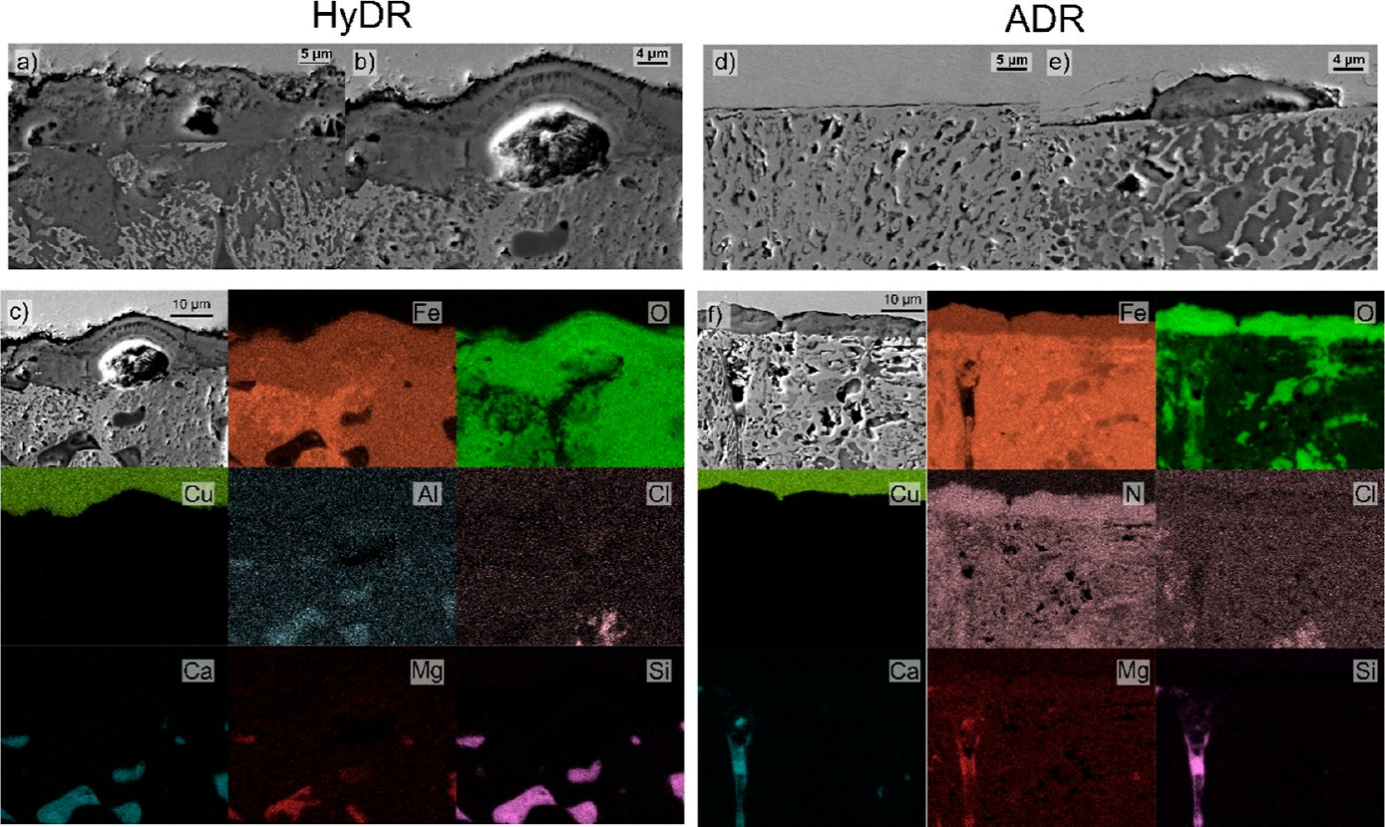
Cross-sectional view of the oxide layer on reoxidized (a,b) HyDR and (d,e) ADR samples. Exemplar chemical mapping of the cross sections is provided in parts (c,f) for HyDR and ADR samples, respectively.

Cross-sectional SEM analysis of the oxide layer and underlying material, presented in Figure 8, provides further insights into the differences in the reoxidation between HyDR and ADR pellets. The HyDR sample displays overall a considerably thick oxide that on average reaches a thickness of about 10 μm, as seen from Figure 8a. Individual portions, corresponding to the regions with a higher presence of Si-, Ca-, and Al-rich oxides and/or pores displaying even higher thicknesses of oxides, related to the evolution of the oxide layer beyond magnetite (see example Figure 8b). The increased oxidation is related to the stronger acidification that occurs within the pores of the material originating from a strong anodic potential formation.^47,48^ The cross sections also provide a clear indication of top-side oxide layer development and growth that is exerted by the oxidation of individual parts of the material from bottom portions of the material and pores, as clearly seen in Figure 8a. The deep penetration of oxidation into the as-reduced pellet material is seen from local chemical mapping with EDS (Figure 8c). It shows that the oxidation of the material reaches several 10 μm deep. The maps also show clearly how the porosity and gangue elements influence the reoxidation dynamics by increasing the corrosion propagation. In contrast, the ADR sample displayed a considerably thinner oxide layer that was on average around 1 μm thick or less, as can be seen from Figure 8d. Similar to the HyDR sample, individual regions showed thicker oxide layers that resulted from increased oxidation related to local porosity variation and gangue element oxides. These sections revealed a similar thickness of the build-up oxide as in the case of the HyDR, but the underlying bulk material in ADR remains less oxidized compared with that of the HyDR sample. The latter can be clearly seen from the chemical mapping of the cross sections of the two samples (compare Figure 8c and 8f). Noteworthy is also the difference in contrast of the Fe maps and the resulting oxidation, which can be linked to the nitrogen-rich material in the ADR sample. The nitrogen enrichments are considered to provide buffering of the local pH through release of nitrogen and/or formation of a nitrogen-rich layer that compensates the anodic state in the porosities through NH_4_^+^ formation that reduces the overall corrosion progress.^47,49^

### Reoxidation Products Probed with Surface-Sensitive Techniques

To provide in-depth identification and understanding of the reoxidation products and their evolution for both the HyDR and ADR samples, Raman shift spectroscopy and XPS were used.

**Figure 9:**
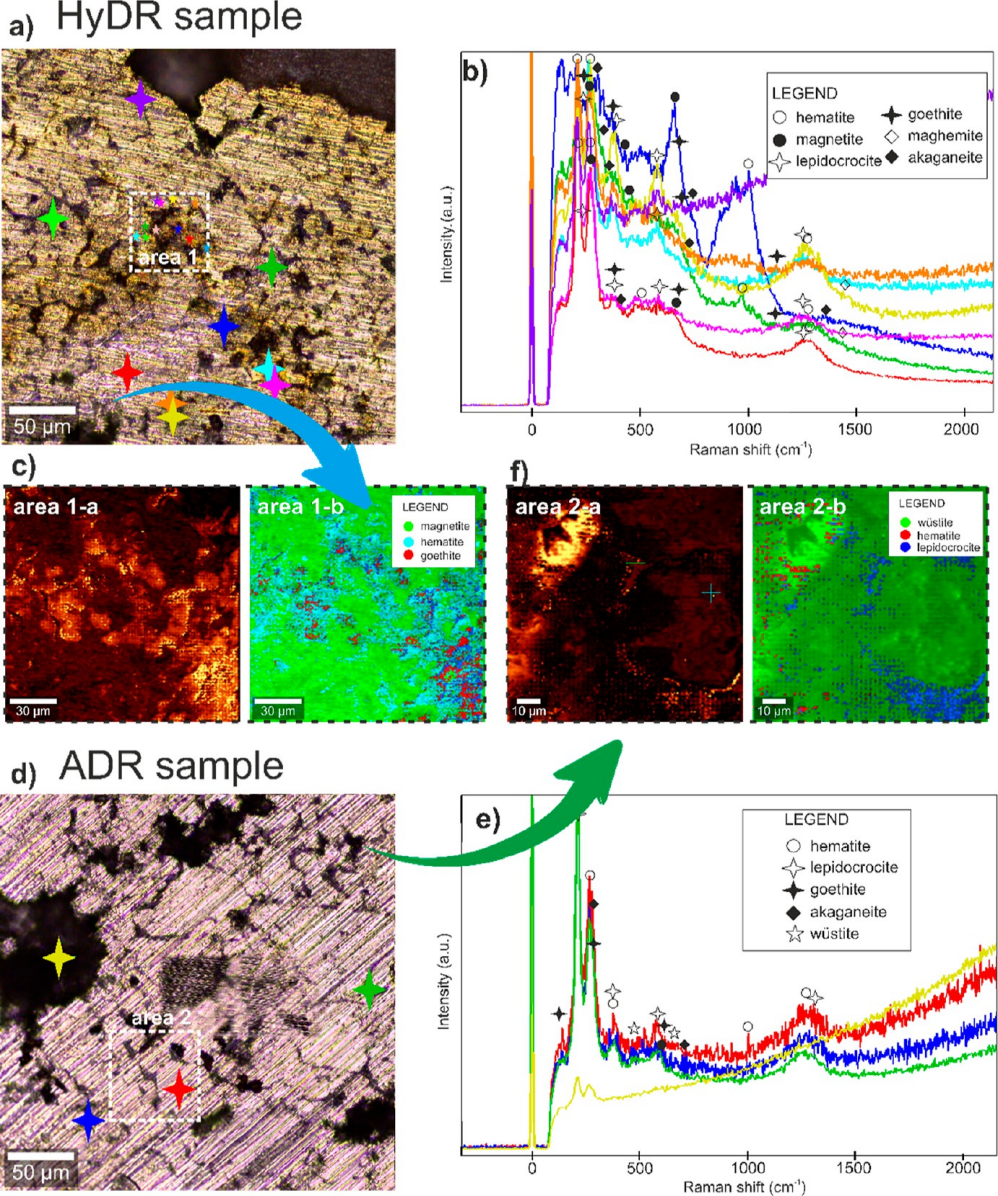
Raman shift spectroscopy data of (a–c) HyDR and (d–f) ADR samples. (a,d) Overview images, where the overall Raman shift spectroscopy was performed. The overall Raman spectra of the HyDR and ADR samples are provided in (b,e), respectively. The individual characteristic shifts of individual oxides are marked in each spectrum with designated symbols. In (c,f), composite Raman shift maps are provided to display the occurrence of the specific shift of selected oxides as defined by the legend. The accompanying images on the left-hand side of (c,f) display the reflected light signal intensity across the analyzed area. The specific positions of analyzed areas are also marked in the overview images (a,d) with colored stars that correspond to the colored Raman spectra from (b,e).

The acquired Raman shift spectra from both HyDR and ADR bulk samples, presented in Figure 9a,b,d,e, exhibited analogous Raman shifts within the range from 100 cm^–1^ to roughly 1300 cm^–1^. Within each sample, the selected area(s) was also mapped to observe the spatial distribution of the iron oxides (Figure 9c,f). The characteristic peaks of hematite (α-Fe_2_O_3_) are present at approximately 225, 290, 490, 510, 660, and 1318 cm^–1^ for the HyDR sample and 218, 280, 400, 1050, and 1318 cm^–1^ for the ADR sample, which correlates well with bulk hematite characteristics.^50^ However, the observation of the 1050 and 1318 cm^–1^ peaks and their curvature in ADR indicate the characteristics of inorganic Vani-type hematite.^51^ To continue, the observations confirm magnetite (Fe_3_O_4_) for the HyDR sample, with characteristic peaks at about 200, 300, and 525 cm^–1^, with a distinguishable peak at 660 cm^–1^. On the other hand, no magnetite was detected in the ADR sample. In the HyDR sample, lepidocrocite (γ-FeOOH) was also detected with specific peaks at approximately 245, 310, 385, 635, and 1290 cm^–1^, while in the ADR sample, the peak at approximately 250 cm^–1^ is the most significant one.^52^ In both samples, the presence of goethite (α-FeOOH) was detected with the peaks at approximately 270, 310, 385, 410, 520, 650, and 1050 cm^–1^ for the HyDR sample and 210, 250, 300, 390, and 580 cm^–1^ for the ADR sample. In both samples, akaganeite (β-FeOOH) was also detected with peaks approximately at 390 and 715 cm^–1^ for the ADR sample, and at 310, 385, 510, 550, and 710 cm^–1^ for the HyDR sample. The most distinguishing difference between the two samples lies in the presence of maghemite and wüstite. The HyDR specimen contains also maghemite with peaks at approximately 380, 450, 520, 675, and 720 cm^–1^. Only in the ADR sample, the presence of wüstite is confirmed by peaks at approximately at 490 and 590 cm^–1^, as has also been observed in other studies on wüstite.^53,54^ The mapping also additionally confirmed the observation by point spectra (Figure 9b,f), where the distribution of the presented iron oxide and hydroxide types can be observed (Figure 9c,f). The correlation between the presence and growth of the iron oxides that form specific features seen from the mapping can be correlated to the free surface energy and size of the oxide types detected with Raman shift spectroscopy.^55^

The XPS results also show a significant difference between the HyDR and ADR samples after exposure to NaCl, with the main difference, as expected, being in the binding energy of nitrogen and oxygen. The overview XPS spectra are provided in Figure S11. In the ADR sample, a number of oxidation states of the following elements can be detected: iron (Fe 2p), oxygen (O 1s), nitrogen (N 1s), silicon (Si 2p), calcium (Ca 2p), and magnesium (Mg 2s). In HyDR, additional peaks from titanium (Ti 2p) and vanadium (V 2p_1/2_) were detected. Due to the exposure of the samples to NaCl, also sodium (Na 1s) and chloride (Cl 2p) peaks were found.

**Figure 10:**
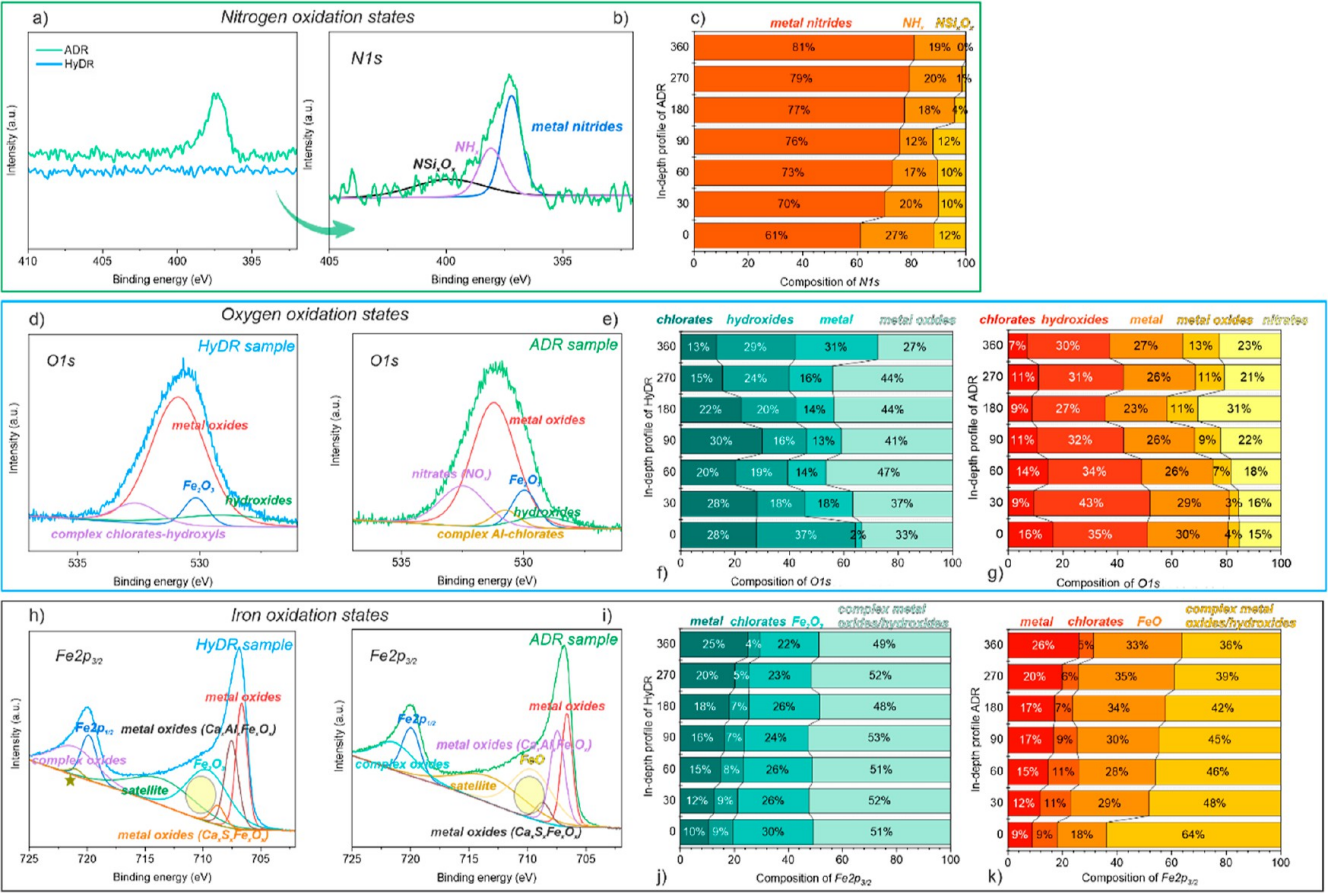
High-resolution XPS spectra of HyDR and ADR samples, where (a) presents the N 1s spectra of both samples (ADR: green line; HyDR: blue line), (b) presents the deconvolution of N 1s for the ADR sample, and (c) presents the in-depth profile of N 1s oxidative-state species for the ADR sample. O 1s deconvoluted spectra for the (d) HyDR and (e) ADR sample. Subfigures (f,g) represent in depth profiles of the oxidation states for both samples, (f) for HyDR and (g) for ADR. Fe 2p_3/2_ deconvoluted spectra, for the (h) HyDR and (i) ADR materials, respectively. The last two graphs (j,k) represent the in-depth profiles of the iron’s oxidation-state species (Fe 2p_3/2_) for both samples, (j) for HyDR and (k) for ADR.

For determination of the different chemical binding states in relation to N 1s, O 1s, and Fe 2p_3/2_, high-resolution XPS spectra were acquired (Figure 10). In HyDR, no nitrogen was observed, whereas in the ADR material, a N 1s peak was detected, Figure 10a. The deconvolution of the broad peak of the N 1s characteristics, given in Figure 10b, revealed two main chemical states in the form of metal nitrides (∼397.1 eV) and NH_*x*_ (∼398.5 eV), which additionally supports the correlation between the different oxides’ buildup in HyDR and ADR samples. The detailed analysis of XPS data is provided in Text S4.

The depth profiles of both samples confirm the observations of Raman shift spectroscopy and surface XPS, which showed the differences among the selected oxides. The depth profile of N 1s showed the presence of nitrogen only for the ADR material in the forms of metallic nitrides, NH_*x*_, and complex Nsi_*x*_O_*x*_. With a longer etching time (and thus depth into the material), the ratio of them changes, with an increase in the metal nitride form of N 1s and a reduced presence of NH_*x*_ and complex Nsi_*x*_O_*x*_ oxides (Figure 10c). The depth profile related to oxygen (O 1s) revealed different compositions of the oxidation states in the ADR and HyDR samples. The comparison of both samples suggests differences in the presence of complex metal oxides as a function of depth from the surface and the presence of nitrates in the ADR sample, which correlates to the presence of nitrogen in the sample. The different ratio among the oxides with progressing depth sensing (see Figure 10f,g) could indicate the local spatial in-plane variation of the oxides as a function of local porosity features and microstructure. The observation also showed the presence of chlorates, correlated with the medium (NaCl) used in the reoxidation experiments. The fraction of chlorates decreases with increasing depth of the sample.

The detailed observation of iron and its oxidation states (Fe 2p_3/2_) also showed the presence of chlorates for both samples in a decreasing fashion from the surface toward the bulk (Figure 10j,k), correlating with the O 1s results. The observation of the HyDR sample showed a similar fraction of complex oxides/hydroxides and Fe_2_O_3_ from the surface to the deeper part of the sample with the presence of the metallic part increasing from the surface to the bulk. A quite diverse distribution of complex metallic oxides/hydroxides is observed in the ADR material on the surface, where most species pertain to either of these two groups (64%). However, with increasing depth, the oxide/hydroxide ratio decreases, and it is partly replaced by the formation of FeO, a scenario which then remains unchanged throughout the depth (Figure 10j,k). The ratio could also be influenced by the presence of nitrogen that can induce the formation of complex oxides and hydroxides, especially in surfaces with high porosity as in the case of the researched reduced material.^56,57^

### Industrial Implications

The results of this study indicate that regulation of the nitriding effect in sponge iron with ADR is possible, which can be performed through a two-step process in order to provide only superficial protection of the reduced pellet from reoxidation and to keep the content of nitrogen low. The first option would be to perform the ADR process in a shaft furnace with a double section, with a NH_3_-rich section for the reduction process followed by an NH_3_-depleted gas mixture section for the gradual cooling and controlled limited nitriding. The other option would be to perform a combination of HyDR and ADR through a concentration gradient of H_2_- and NH_3_-rich regions that would provide the direct transit of reduced pellets through the two reduction processes. The latter option is also naturally more easily achieved through strong density separation of the gases and decomposition products of NH_3_ gas and H_2_ gas. Additionally, the recuperation of H_2_ derived from ammonia decomposition could be more easily achieved through the accumulation of H_2_ in the upper part of the shaft furnace, thus separating from the nitrogen-rich zones that would primarily be concentrated in the lower part of the shaft furnace. Through the nitriding process and the many options to tune and control it, the ADR process provides possibilities to obtain high-grade sponge iron with sufficient nitrogen incorporation for reoxidation protection, required for downstream handling and logistics, which averts the need for hot briquetting, cutting down processing costs, leading to a new pathway toward sustainable and CO_2_ reduced production of iron and steel.

## Conclusions

In this study, the temperature dependence of the ADR of commercial hematite pellets has been investigated with respect to kinetics, phase formation, elemental partitioning, nitriding, nitrogen solubility, microstructure, and heterogeneity. The results indicate that the reduction progresses via oxygen removal by the ammonia decomposition products, i.e., hydrogen gas, as well as through incorporation of solute nitrogen into the material. At temperatures below 600 °C, in situ nitriding accompanies the reduction process, forming predominantly Fe_3_N-type nitrides, and this process becomes more dominant at lower temperatures. The nitrides form in an inhomogeneous manner that is governed by the local concentration of ammonia gas and its decomposition products as well as by direct nitriding of the material via diffusion into both iron oxides and iron domains. At higher temperatures above 600 °C, the ammonia decomposition becomes more prominent and results in fast kinetics of the ADR and retardation of in situ nitriding due to the thermal instability of the nitrides, resulting in reduction kinetics similar to that of HyDR. Upon cooling in ammonia, the pellets display spontaneous nitriding, resulting in the dominant formation of Fe_4_N. However, even with cooling in non-nitriding media (e.g., Ar), the ADR process still yields nitrogen incorporation, albeit with a lower degree, related to the diffusion of nitrogen from the surface to the bulk of the pellet material through intermediate partial in situ nitriding that becomes instable with progressing reduction time. The heterogeneity of the initial pellet structure and its porosity also play a fundamental role in the development of the reduction and nitriding of the material. The incorporation of nitrogen into the ADR pellets as both nitrides and solid solution provides superior protection against reoxidation. When comparing ADR against HyDR, it displays considerably slower development of the oxide layer as well as reduced penetration of oxygen into the bulk part of the reduced pellet. Overall, this study provides important insights into the basic underlying mechanisms and kinetics of in situ and spontaneous nitriding. These observations are required for understanding and optimizing the ADR process with respect to reductant gas mixtures, microstructure, and chemistry at different temperatures. However, a deeper investigation is still required, particularly in connection to understanding the local nitriding effects and limitations associated with local chemistry, porosity, and accessibility to the reduction gas. Potential opportunities lie in analyzing these questions using sophisticated in situ probing setups with high spatial and temporal resolution that can probe materials directly during the reduction process or through an environmental cell.

## Data Availability

All data are available in the main text or the Supporting Information.

## Supporting Information Available

Discussion on pros and cons of using different hydrogen carriers for direct reduction; theoretical discussion of ammonia reduction and nitriding; water signal discussion; XPS data analysis; additional data of gas evolution and reduction kinetics; macroscopic images of sample and cracking; XRD diffractograms of ADR samples reduced at 500 °C; chemical composition profiles extracted from XRD data; SEM and EDX images of the ADR sample reduced at 550 °C for 4 h; reduction degree comparison with cooling in NH_3_ and Ar gases; cross-section XRD data of the ADR sample reduced at 700 °C for 2 h; XRD diffractograms displaying chemically induced peak shifting; SEM and EDX images of the ADR sample reduced at 600 °C for 2 h and cooled in Ar gas; overview XPS spectra of ADR and HyDR samples; and XRD measurements of sample surfaces reduced at different temperatures and cooled in Ar gas (PDF)
