# Supplementary material for: Thermal Kinetics and Nitriding Effect of Ammonia-Based Direct Reduction of Iron Oxides
Source: ACS Sustain Chem Eng. 2024 Jun 17;12(26):9882–96. doi: 10.1021/acssuschemeng.4c02363 (PMC11220788; doi:10.1021/acssuschemeng.4c02363)
Supplement: Supplementary file 1 — sc4c02363_si_001.pdf [file sc4c02363_si_001.pdf]

Thermal kinetics and nitriding effect of ammonia-based direct reduction of iron oxides

Matic Jovičević-Klug[1,], Yan Ma[1,*], Patricia Jovičević-Klug[1], J. Manoj Prabhakar[1], Michael Rohwerder[1], Dierk Raabe[1]*

[1]Max Planck Institute for Sustainable Materials (new name); 40237 Düsseldorf, Germany / Max-Planck-Institut für Eisenforschung (old and legally binding name); 40237 Düsseldorf, Germany.

2 Alexander von Humboldt Research Fellow, Alexander von Humboldt Foundation, Jean-Paul-Straße 12, 53173 Bonn, Germany

* Corresponding author: m.jovicevic-klug@mpie.de, y.ma@mpie.de

Content:

29 pages

4 Texts

12 Figures

Text S1 Discussion on pros and cons of using different hydrogen carriers for direct reduction

With the emerging projects and EU strategic plans, such as REPowerEU, the EU aims to significantly reduce the CO_2 emissions from the steel industry by replacing around 30% of primary steel production with hydrogen-based direct reduction (HyDR)[1]. However, HyDR comes with many challenges related to both production and storage of hydrogen. More specific, the industry is particularly facing challenges in the on-site production of hydrogen as the transport of hydrogen is considerably energy intensive and inefficient due to the high pressures of up to 700 bar or low temperatures of -253 ˚C required for hydrogen liquification that alone can consume up to 6.451 kWh per kg of hydrogen[2]. In practice, however, the energy consumption for the entire liquefaction, transfer, and filling process (with losses of 5-10%) can be as high as 13.3 kWh per kg of stored liquid hydrogen, which is about 33% of the gross calorific energy of hydrogen (40 kWh/kg). Furthermore, due to the large temperature differences and high pressures, the transport and storage containers have considerable boil-off losses. When using new storage technology this loss can be reduced to below 0.8 % [3] and as low as 0.4 to 0.06 % [4] (depending on storage capacity) per day for transport and storage, respectively. Nevertheless, the values clearly depict that long-term storage of hydrogen in large quantities cannot be trivially addressed, which can pose a considerable challenge for steel industries without local hydrogen production.

Alternatives are being sought to overcome the problems associated with the energy-intensive transport and storage of hydrogen. One solution is to use methane as a hydrogen carrier

for the reduction process, either by converting it to hydrogen and carbon monoxide on site just before the reduction process, or by using it directly in the reduction process. Methane is much easier to transport and store than hydrogen. While the use of methane still results in CO_2 emissions, the values are significantly lower, reaching about 1.1-1.2 tonnes of CO_2 per tonne of steel, when methane is for example used directly in a steam methane reforming unit that produces the reductant feedstock such as in the MIDREX process[5]. If the conversion of hydrogen is performed directly and with appropriate CO_2 removal via the production of so-called blue hydrogen, the CO_2 emissions can be lowered down to 8.5 kg CO_2 per kilogram of hydrogen gas[6]. The reduction of iron ore to 1 tonne of iron requires approximately 54 kg of hydrogen[7] when assuming 1:1 stoichiometric conversion. However, due to the additional hydrogen requirement for accommodating heating and losses, the hydrogen consumption is projected to be around 60 kg per tonne of liquid iron[8]. This means that with the methane route only 0.5 tonne of CO_2 per tonne of steel would be produced, which is a reduction of nearly 4-fold compared to the conventional blast furnace route. The main challenge of using methane in this way is that it would require additional on-site facilities at each steel plant, making it a CAPEX (capital expenditure) intense process. The main disadvantage of using methane is the necessity to use mining/gas extraction or fossil-fuel derivation processes, which add negatively to the impact of the reduction process on the environment[9].

In contrast, ammonia can be easily transported and due to the existing infrastructure, which originates from ammonia transport for fertilizer and chemical production, the transition to this medium for steel production would be relatively straightforward. As presented in several papers,

ammonia, owing to its relatively high boiling point[10], can be stored in liquid form under mild conditions of 8 bar at 25 ˚C or at 1 bar at -33 ˚C thus delivering high hydrogen density[11]. This brings ammonia at the forefront of gases that can be used to transport and produce hydrogen[12]. However, methanol has even better characteristic in terms of liquification and energy density[12] and is also considered as one of the alternatives for fossil-free production of direct reduced iron[13]. However, the utilization of methanol, unlike other hydrogen carriers, cannot be performed directly, but it is necessary to reform the methanol outside of the direct-reduction furnace to produce proper reducing gases[13], making such process technologically difficult and expensive. Additionally, while methanol is much safer in terms of storage, the implications through the lower combustion efficiency, inherent containment of oxygen, corrosiveness, risk to human health and its inherent CO_2 footprint give technological challenges for its utilization in large-scale industries[14] to reduce their carbon footprint.

In terms of utilization of ammonia for reducing the impact on global warming also the formation of NO_X needs to be carefully assessed. It is well known that NO_X generation with ammonia is particularly high, when the combustion is not complete or when combustion is performed in oxygen-rich environment[15,16]. However, the NO_X generation can be reduced through optimized and properly regulated processes or through subsequent conversion processes such as De-NO_x[16]. However, further studies are required to confirm the level of NOx generation in the solid-state reduction of iron oxides, especially in terms of understanding the catalytic effect of iron oxides and formed iron[9]. A potential to combat the different shortcomings of individual hydrogen-carrying gases also lies in utilizing gas mixtures that in combination can reduce the individual

toxicity, environmental impact and safety risks, while improving energy density, combustion and handling of them[17,18].

On the other hand, ammonia possess an inherent toxic character to the environment and humans, creating challenges in its handling and usage[14]. However, technology for mitigating these risks have been established owing to the density of liquid ammonia[11], easy detection of it in trace amounts in ppm range[19–21], fast dilution with water[10] and its long-term storage stability and low leakage rates[12]. In terms of ignition safety and fire hazard as well as the overall safety of the different hydrogen carriers, ammonia stands as one of the safer gases due to its low risk of explosion and combustion characteristics, such as flammability limit and minimum ignition energy[22]. Overall, ammonia with its including risks related to its inherent toxicity lies as one of the safest alternative hydrogen carriers for use in direct reduction processes[14,22].

Text S2 Theoretical discussion of ammonia reduction and nitriding

At higher temperatures, such as at 700 ˚C, the ammonia readily decomposes into nitrogen and hydrogen gases, as per Equation 1. Then, the hydrogen directly being consumed for the reduction of the nearest iron oxide surface as in HyDR [23] and the reactions follow the same pathway as presented by Equations 2,3 and 4, that progressively results into formation of iron through solid state reduction. The remaining nitrogen species on the surface have a lower potential to form strong Fe-N bonds and corresponding nitrides with the initial oxide material and thus only dissociate as gaseous species rather than stabilizing into the NH^+ and NH_2^+ species on the reducing oxide surface

or formed iron[24]. As a result, at higher temperatures in-situ nitriding is thermodynamically unfavourable and results in release of N_2 gas.

(1) $$2 \cdot NH_3 \xrightarrow[Fe\ oxide]{} N_2 + 3 \cdot H_2$$

(2) $$3 \cdot Fe_2O_3 + N_2 + H_2 \xrightarrow[T \geq 600\ °C]{} N_2 + 2 \cdot Fe_3O_4 + H_2O$$

(3) $$Fe_3O_4 + N_2 + H_2 \xrightarrow[T \geq 600\ °C]{} N_2 + 3 \cdot FeO + H_2O$$

(4) $$FeO + N_2 + H_2 \xrightarrow[T \geq 600\ °C]{} N_2 + Fe + H_2O$$

At lower temperatures of e.g. 500 ˚C the full ammonia decomposition is significantly retarded, which originates from the higher thermodynamic favourability of Fe-N bond formation and nitrides. It can thus be postulated that the initial ammonia effectively nitrides the material and subsequently removes oxygen through a two-step process with the initial dehydrogenation forming the NH_2^+ species (see Equation 5) that later dissociates through direct reaction with hematite to form magnetite and then iron nitride according to Equations 6 and 7. This step is considered to occur in a dimer fashion as described by Yeo et al[25]. for the ammonia decomposition on iron. For presentation purposes the reactions with NH_2^- are provided in Equations 6-8. As can be seen from equations 5-8, the electron imbalance indicates the requirement of addition or removal of electrons that relate to the limitation of the nitriding reactions by the dissociation of the oxygen from the oxide and decomposition of the ammonia. This means that nitriding at high temperatures does not occur spontaneously, but forms through sub-processes which require the presence of hydrogen and (partially) reduced iron oxides and metallic iron as given by the described set of equations. In other words, nitriding does not occur directly via N_2 (*e.g.* via complete dissociation of ammonia), but

rather through the ammonia decomposition sub-products such as NH_2^- and NH^{2-} or more complex arrangements of these with the reduced material[25].

The dimeric ammonia reduction also explains the low remaining amount of FeO in the reduced material, due to balancing effect of the reaction as seen by comparing Equation 5 and 8 that even out the charge imbalance in relation to the surplus and depletion of the additional 2 electrons.

(5) $$2 \cdot NH_3 + 2 \cdot e^- \xrightarrow[Fe\ oxide,\ T \leq 550\,°C]{} 2 \cdot NH_2^- + H_2$$

(6) $$3 \cdot Fe_2O_3 + 2 \cdot NH_3 + 2 \cdot e^- \xrightarrow[T \leq 550\,°C]{} 2 \cdot NH_2^- + 2 \cdot Fe_3O_4 + H_2O$$

(7) $$Fe_3O_4 + 3 \cdot NH_2^- + H_2 \xrightarrow[T \leq 550\,°C]{} 3 \cdot FeN + 4 \cdot H_2O + 3 \cdot e^-$$

(8) $$2 \cdot FeO + 2 \cdot NH_2^- + H_2 \xrightarrow[T \leq 550\,°C]{} 2 \cdot FeN + 4 \cdot H_2O + 2 \cdot e^-$$

(9) $$Fe_2O_3 + 2 \cdot NH_3 \xrightarrow[Fe\ oxide,\ T \leq 550\,°C]{} 2 \cdot FeN + 3 \cdot H_2O$$

The reduction and nitriding must be described in relation to the formation of Fe_3N that is thermodynamically stable and preferred at lower temperatures as observed from experimental results. For these purposes the direct routes from hematite (Fe_2O_3) are discussed for ease of explanation.

(10) $$3 \cdot Fe_2O_3 + 6 \cdot NH_3 \xrightarrow[T \leq 550\,°C]{} 2 \cdot Fe_3N + 9 \cdot H_2O + 2 \cdot N_2$$

As seen from Equation 10, the reaction can balance out by the production of nitrogen gas with the formation of nitride from hematite. However, as seen from previous Equations 6-8 the oxide will probably develop the nitride to the stepwise reduction of the oxide rather than through the direct change of the oxide to nitride. For this reason, it is postulated that the nitrogen incorporation at high temperatures must occur through the solid solution of nitrogen into the reduction sub-

products of the iron oxide and later of the iron and/or metastable FeN, which cannot be directly described through chemical reactions. This can also explain the nitrogen enrichments seen in the samples, even when no nitrides were detected (see experimental results for more details).

Nitrogen diffusion is the limiting factor for the spontaneous nitriding event. The literature indicates that the diffusion and solubility of nitrogen in iron is low[26,27], reaching at most 450 ppm[28]. However, the nitriding rate for iron at 1 bar of NH_3 is also slow[29], indicating that concentration-gradients in oxygen and nitrogen (as well as of the ammonia decomposition sub-species) might provide the locally higher driving forces to exceed the thermodynamic bulk solubility limits of nitrogen in iron. Previous studies have shown that iron can, in principle, be supersaturated in nitrogen[30]. However, in relation to ammonia-reduced iron oxide the supersaturation remains an unanswered point here. On the one hand the gas analysis and reduction rates clearly indicate that the ammonia does not completely decompose at lower temperatures, which directly affects the weight loss with progressing reduction and in-situ nitriding. On the other, the spontaneous nitriding clearly presents the correlation to the concentration of the ammonia and nitrogen in the system as the main driving mechanism for the nitride formation, which is also dependent on the reduction temperature. Additionally, from the local chemical mapping and chemical assessment via XRD (see Figures 3-5 and Supplementary Figures S5-S6 and S8-S9) the nitrogen incorporation through the reduction process must occur, but most probably at different degrees based on the temperature and density of the material (related to porosity and access to reduction gas). To clearly present the

effect of temperature on the nitrogen incorporation during the reduction and without the effect of spontaneous nitriding (i.e. cooling in Ar), the outer surface of reduced pellets was also analysed with XRD. This allows access to the direct reaction interface of the reduced material to gain access into possible nitrogen incorporation in form of nitrides. The results, presented in Supplementary Figure S12, provide direct evidence that at lower temperatures of 550 ˚C (performed for 2 h) the nitriding of the surface is considerable (about 55 wt.% of the surface is in form of nitrides).

The remaining portion is in the form of oxides, more specific, mostly in the form of wüstite (FeO) and magnetite Fe_3O_4), which have a similar fraction, with a slight (4 wt.%) presence of hematite. The residual oxides are most likely a result of the fast re-oxidation of the freshly formed iron. Interestingly, the nitrides found on the surface reveal varying crystal structures ranging from FeN, Fe_2N and Fe_3N, without any Fe_4N detected. The presence of the nitrides clearly indicates that the decomposition of the ammonia is complex at low temperatures and the nitriding is not a single step reaction. The presence of instable FeN and Fe_2N, even when cooling in Ar, indicates that the reduction and the nitriding are influenced by the nitrogen diffusion, which must be a key feature for the nitrogen incorporation into the iron as well as partially into the oxide species, namely Fe_3O_4 and FeO that allows the formation of intermediate nitride states. Through this, it is also clear that metastable nitrides are indeed forming on the interfaces, that can thus propel the development of the nitrides through sequential nitriding as per Equation 11. However, as the progression of nitriding is time dependent it is also clear that Equation 11 only explains the simplistic reactions at the direct interface and not when nitrogen is exchange with the bulk of the iron oxide or iron material. This also can mean that despite the tendency of formation of N_2 gas, per Equation 11, a

possible side reaction in form of nitrides or nitrogen incorporation could be present, however, this is only a postulation.

(11) $$Fe_2O_3 + FeN + 2 \cdot NH_3 \xrightarrow[T \leq 550\,°C]{} Fe_3N + N_2 + 3 \cdot H_2O$$

Translating the above behaviour, we can also explain the development of both nitrides and iron in the same process by integrating the development of both phases from Fe_2O_3 and NH_3 as per Equation 12. To this end this would possibly explain the branched development of the nitrides and formation of iron and nitride rich domains in a side-by-side manner as seen from SEM images (see Figure 3 and Supplementary Figure S6) as well as the not complete nitride formation as seen from XRD of the surface of the pellet from Figure S12.

(12) $$4 \cdot Fe_2O_3 + 8 \cdot NH_3 \xrightarrow[T \leq 550\,°C]{} 2 \cdot Fe_3N + 2 \cdot Fe + 3 \cdot N_2 + 12 \cdot H_2O$$

When the surfaces of the samples are reduced at 600 ˚C and 700 ˚C for 2 h (see Supplementary Figure S12), the nitrides are found only in small quantities and only in the form of Fe_3N and FeN (total amount of 2-3 wt.%). The surfaces of both samples are mainly constituted by magnetite and hematite, which – according to the time of the reduction – must be a result of the fast re-oxidation of the newly formed iron, because the samples were fully reduced for the given reduction conditions as indicated by the TGA measurements. The presence of only a few wt.% of nitrides and lack of Fe_4N confirms that the nitriding of the material in form of Fe_4N is mainly due to the fast and spontaneous nitriding effect under presence of ammonia during cooling. However, these results indicated that in-situ nitriding and thus incorporation of nitrogen in the material is present even during the reduction. The possibility for this might lie in the inhomogeneous substructure of

the reduced material coupled with substantial local thermal and chemical gradients that provide further heterogeneity and non-equilibrium conditions for nitriding. In this regard, it is also clear that the reduced volume fraction of the nitriding, and thus nitrogen uptake, with higher temperatures (as seen from Table 1 by comparing 600 ˚C with 700 ˚C and 800˚C examples) is a result of the internal sintering (seen from Figure 6) of the material that prohibits the access of the ammonia gas into the sintered parts of the material that would otherwise increase the nitride concentration in the material during cooling. The present study suggests that most of the nitriding can be avoided, when the pellets are reduced at temperatures of 600 ˚C and above and cooled down without presence of ammonia (e.g., in Ar gas in this study).

Text S3 Water signal discussion

During the reduction, water is produced due to the reaction of hydrogen gas with the oxide material. Occasionally, individual samples reveal stochastic formation of spikes in the water signal (see blue curve in Figure 1b) in the range of 15 min to 1 h after the reduction temperature is reached. The water spiking originates from macroscopic cracking events of the sample during the reduction, which creates large new areas for reduction as well as releases potentially entrapped water vapour, which is formed during the solid-state reduction to iron[31]. Due to the time range, the events are considered to originate primarily from the initial transformation of hematite to magnetite and wüstite that develop volumetric changes originating from the phase transformation and oxygen removal[32]. The volumetric mismatch of the reduced outer part of the samples creates localised stresses that induce localised cracking from the surface to the bulk of the pellet sample[33]. However,

the large spike should be constituting a large-scale event and a possible reason for this could be the prior heterogeneous state of the pellet material inherited from the preceding sintering process[34]. The major cracking event could be in-fact the separation of the more condensed central portion of the pellet from the less dense outer portions that are formed by the prior sintering process. An example of the cracked internal portions of the pellet material is presented in Supplementary Figure S2, which shows clearly that strong cracking occurs between the previously described regions that seemingly form around the entire core of the pellet.

Further support for the formation of new reaction surface is also given by the H_2/N_2 ratio that clearly shows a drop associated with the sudden higher consumption of hydrogen gas that is disproportionate to the general concentration decrease of all the gases with higher water concentration. The ratio of H_2O/H_2, presented in Figure 1f, further shows that the hydrogen consumption per water formation is indeed higher by orders of magnitude, which further support that new reaction interfaces for the ammonia decomposition and iron oxide reduction have formed in this short amount of time. Once the initial reaction and reduction of the new free oxide surfaces is complete, the formerly steady reduction process is reformed and stabilized to a similar value as before the cracking event as can be seen from H_2O/H_2 ratio, as seen from Figure 1f and its insert.

Another aspect on the reduction efficiency with ammonia is given once more by the tracked water signal. As seen in Figure 1b, after approximately 1 h of reduction, the water signal shows a sudden drop and subsequently the water signal continuously drops further. The sudden change in the water signal behaviour is not an artefact as illustrated by the presence of it for all samples as seen from Figure 1f and Supplementary Figure S1. The reasoning for such a drop is the formation

of a continuous, typically hundreds of nanometres thick, iron layer with the reduction that halts the direct release of water from the iron pellet free surface, much like what has been proposed for the HyDR. As a result, the water formation is then limited by the slow diffusion of oxygen through the iron layer to the exposed surfaces and interfaces, which continuously becomes slower with further progression of the reduction. Interestingly, the occurrence of the step in the water signal is not distinctly dependent on temperature, but it seems to be rather related to the individual pellets. As can be seen from Supplementary Figure S1 for the case of the 2 h reduced samples at 600 ˚C (Supplementary Figure S1d-e), the two pellets have different final reduction degrees, with one reaching nearly 10 % lower reduction degree. The differences originate from the much faster reduction degree of the first pellet that is possibly governed by the higher mechanical instability of the pellet, *i.e.* cracking, which correlates with the higher number of water signal spikes as monitored by the mass spectrometry probing (see Supplementary Figure S1d-e). The higher instability of the pellet can thus explain both the faster reduction kinetics as well as the later formation of the water step compared to the other pellet that displayed a more stable behaviour and thus earlier formation of the step in the water signal. Despite the above-described variations from pellet to pellet, the water signal through its general trend and relationship with the reduction degree can be potentially used as a tracer for the reduction degree. It is postulated that this is possible when statistical probability and variation in the pellets is considered, as then the stochastic nature of the abrupt changes in the water signal will even out with large mass integration, whereas the overall proportional decrease of the water signal with reduction degree will be conserved due to the similar trend from pellet to pellet. As such water signal monitoring presents a unique option

for regular tracing and probing of the reduction degree, when the corresponding modeling of the spatial distribution of pellets, temperature and gas composition is properly evaluated in a multi-pellet ensemble and connected with the water signal on a large-scale shaft furnace.

Text S4 XPS data analysis

The oxygen peak *O1s* (see Figure 10d-e) for both samples showed the chemical states of specific metal oxides (~531 eV for ADR, ~530.8 eV for HyDR), more precisely the presence of Fe_2O_3 (~529.9 eV for ADR, ~529 eV for HyDR), hydroxides (~531.2 eV for ADR, ~529 eV for HyDR) and complex chlorates/hydroxyls[35] (~530.7 eV for ADR, ~530.1 eV for HyDR). In the ADR sample, the presence of various nitrates species (~532.47 eV) is observed. The $Fe2p_{3/2}$ peak (Figure 10h-i) for both ADR and HyDR samples shows also the presence of metallic Fe (~706.6 eV ADR, ~706.7 eV HyDR), complex metal oxides (~707.4 eV ADR, ~707.7 eV HyDR) that could be formed by Ca, Al and Fe[36] and an additional peak (~708.6 eV ADR, ~708.7 eV HyDR), which could indicate complex metal oxides in both samples formed from Ca, Fe and S[37]. The slight shift of the metallic oxidation peaks in the ADR material can also indicate the complex nature in combination with nitrogen (Fe_xN_y)[38], corresponding well to the inherent presence of nitrides in the ADR sample. In the ADR sample also more pronounced shifts related to wüstite (FeO) at around 709.7 eV are detected, which is aligned with the findings of EDS and Raman. The peak of wüstite/Fe_2O_3 partly overlaps with the peak of Fe-chlorites (~710-710.4 eV), since the samples were exposed to NaCl (marked by the yellow region in Figure 10h and 10i). In the HyDR specimen the presence of Fe_2O_3 (~710.3 eV) was determined as well as an additional peak at 721.5 eV

(marked with star symbol in Figure 10h), which could be correlated to the degradation of Fe into the Fe(II)/Fe(III) form[39].

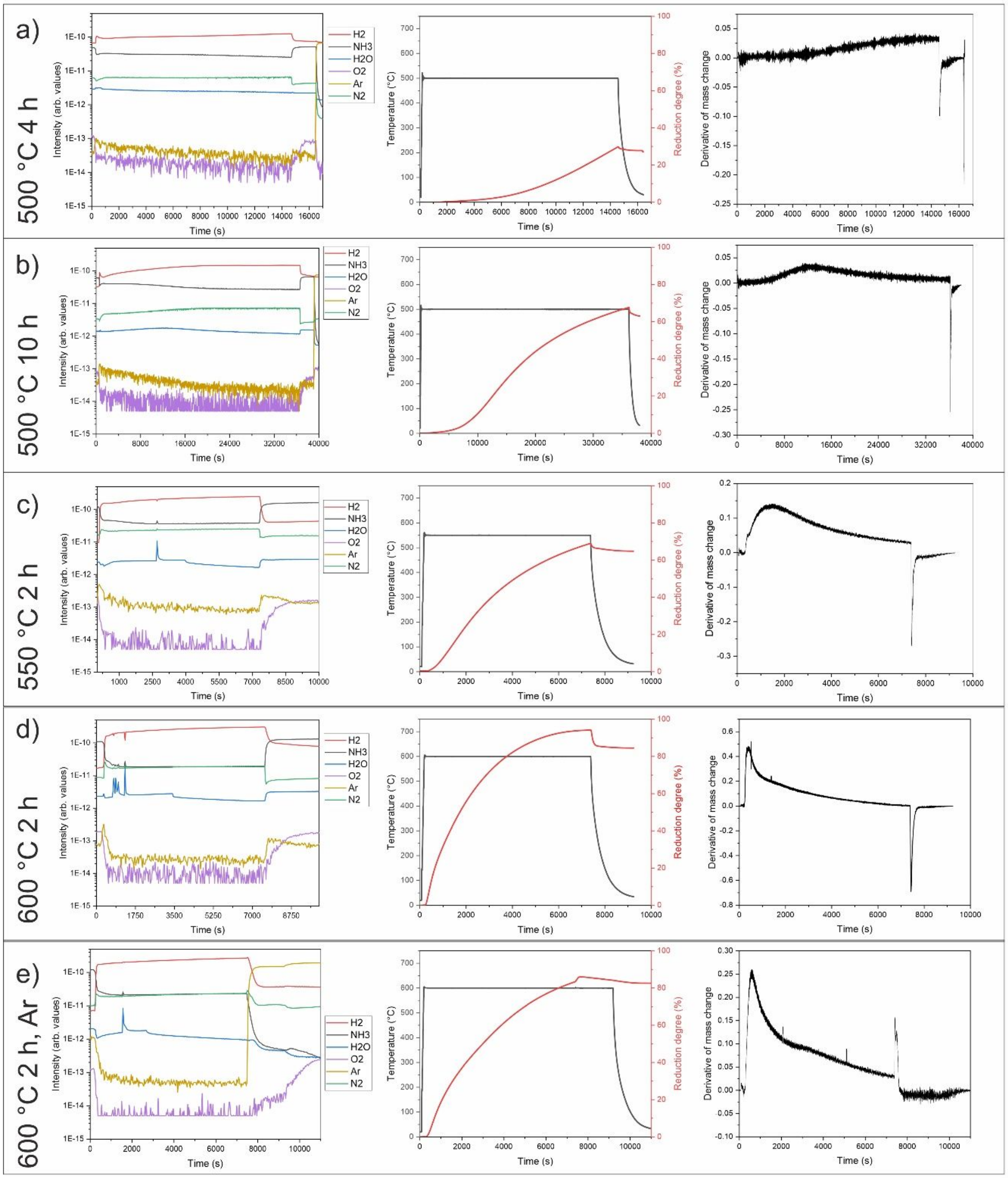

Supplementary Figure S1: Gas evolution obtained from in-situ measurements with a mass spectrometer, mass change and temperature evolution with reduction time and the directive derivative of the mass change during ADR samples at a) 500 ˚C 4 h, b) 500 ˚C 10 h, c) 550 ˚C 2 h, d-e) 600 ˚C 2 h. All the samples were cooled in ammonia gas, except for e, which was cooled in Ar gas.

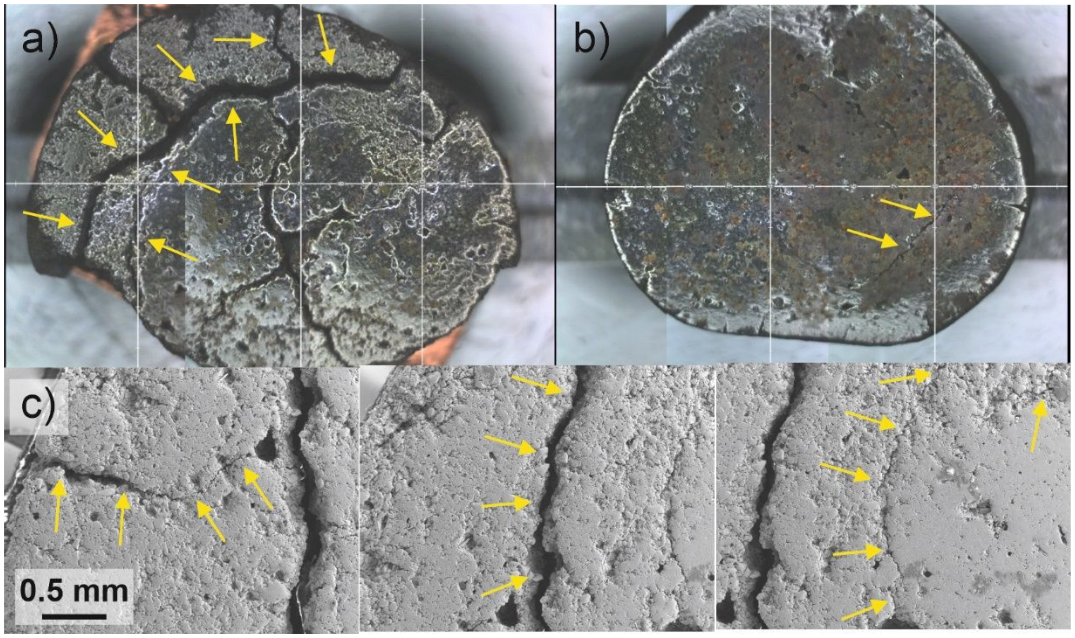

Supplementary Figure S2: Macroscopic images of cracking phenomena observed for ADR samples reduced at a) 550 ˚C for 4 h and b) 600 ˚C for 2 h. Note that due to the higher nitriding effect on the 550 ˚C, the cracking of the samples is stronger due to the higher brittleness of the

nitride phases. c) scanning electron micrographs displaying the larger cracks forming within the pellet material in a radial form that predominantly encircle the core of the pellet. The yellow arrows in all the images indicate the cracks that are presumed to originate from the reduction of the oxide material.

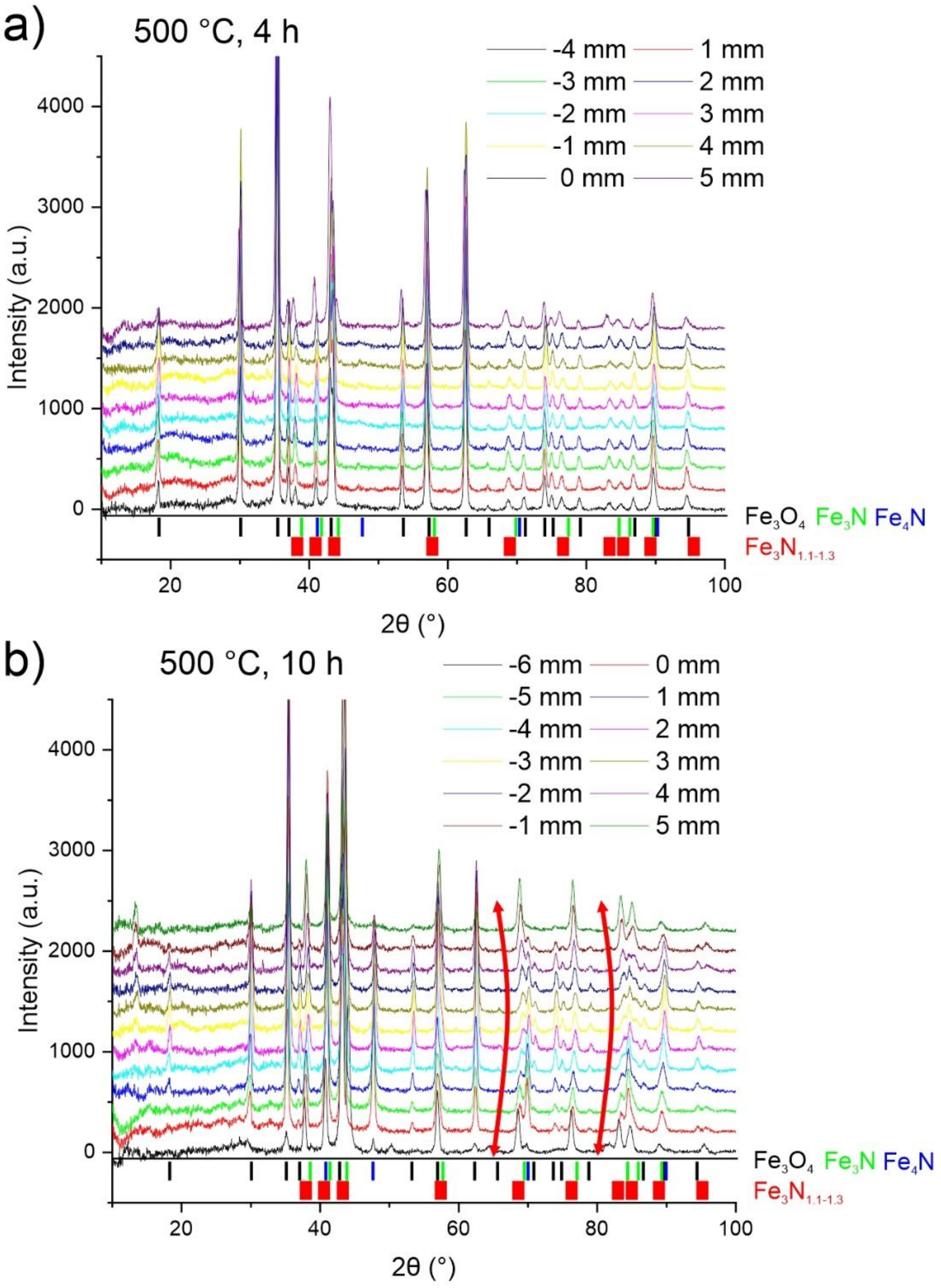

Supplementary Figure S3: XRD diffractograms of the cross-sectional measurements of the ADR samples reduced at 500 ˚C for a) 4 h and b) 10 h. The diffractograms are presented in a waterfall form from one edge to the other of the pellet's cross-section (denoted with negative and positive

distances from the centre of the pellet). The identifying cards of the major phases identified in all of the diffractograms are provided below the individual waterfall stack.

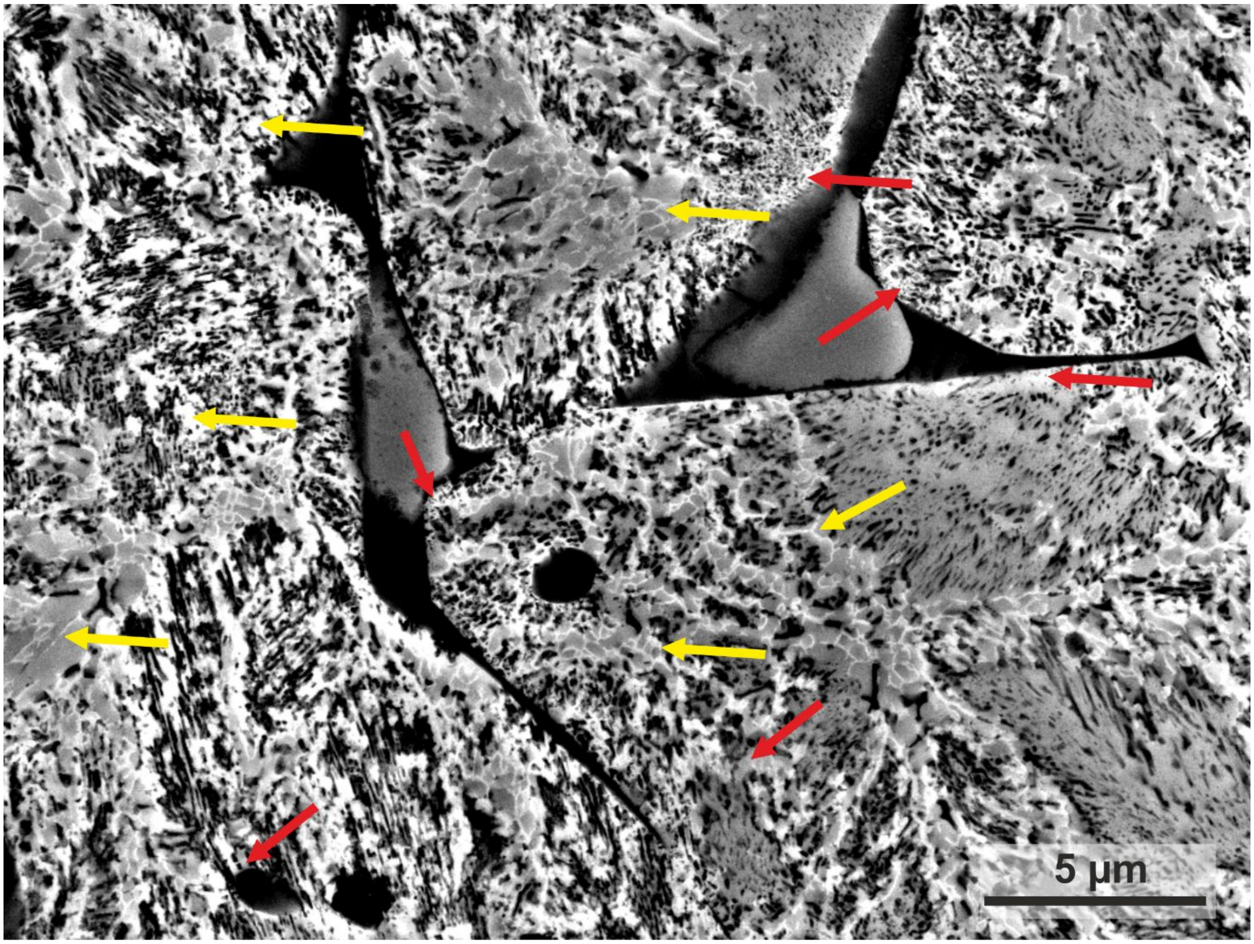

Supplementary Figure S4: Secondary electron image of a middle portion of the ADR pellet reduced at 500 ˚C for 10 h. The red and yellow arrows indicate the different nitrides types, namely Fe_3N and Fe_4N, respectively.

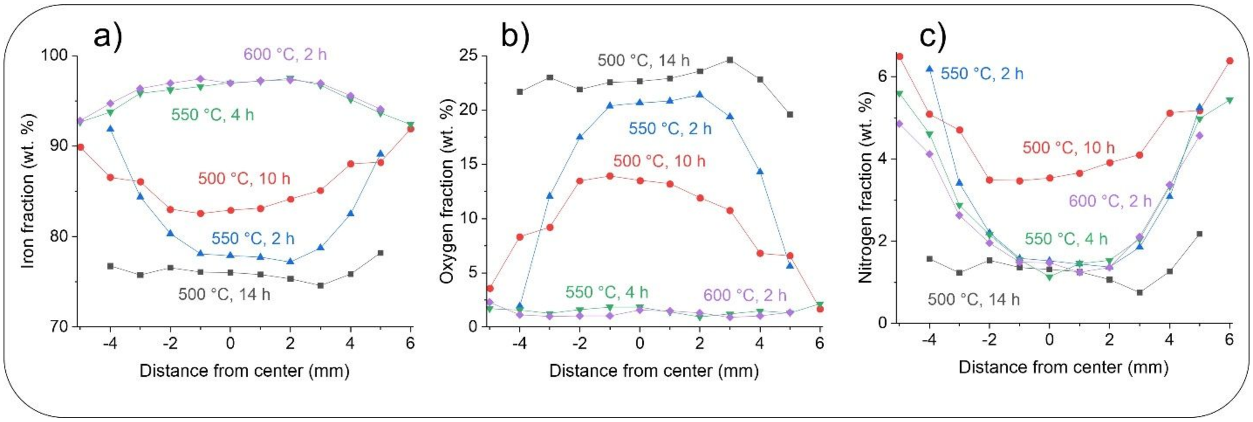

Supplementary Figure S5: Extracted chemical composition profiles of the cross-sections for a) iron, b) oxygen and c) nitrogen of the ADR pellets reduced under different temperatures and for different times. For the calculations to extract the chemical profiles, the nominal chemical composition of the identified phases from XRD analysis was used and summed-up to derive the weight fraction of individual elements.

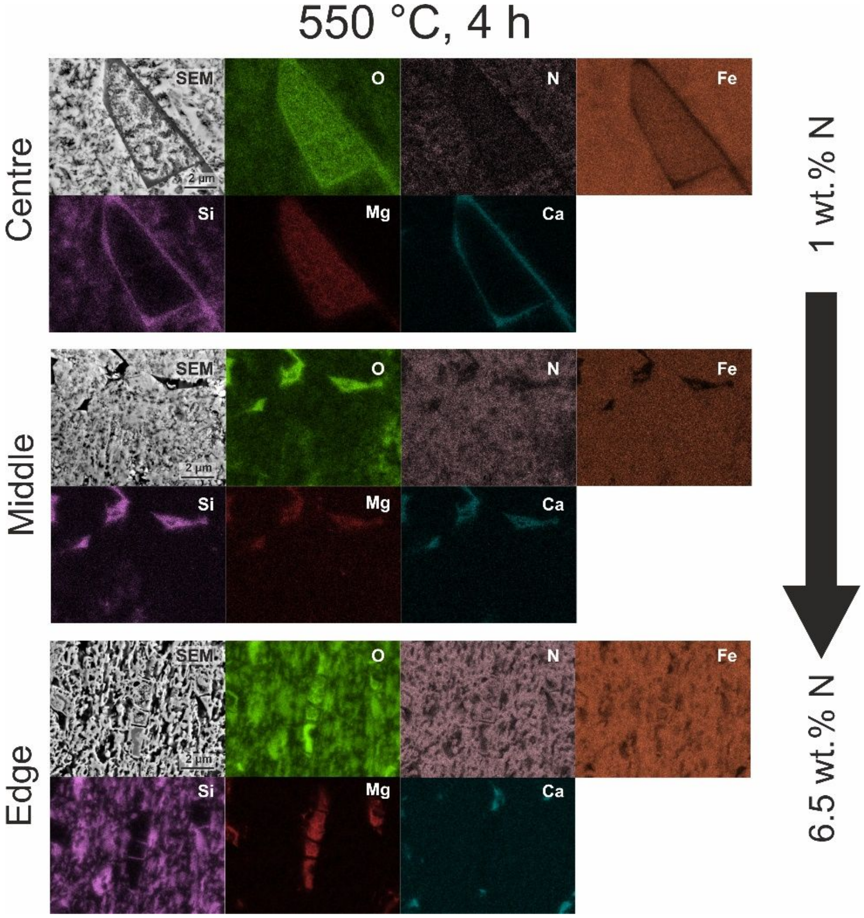

Supplementary Figure S6: Secondary electron images (SEI) and elemental distribution maps obtained with electron dispersion X-ray spectroscopy (EDX) for ADR pellet reduced at 550 ˚C for 4 h. The different regions represent a typical microstructure present in the different portions of the pellet. The accompanying maximum and minimum values of nitrogen situated next to the arrow is acquired with local EDX quantitative analysis in the differently specified regions of the pellet.

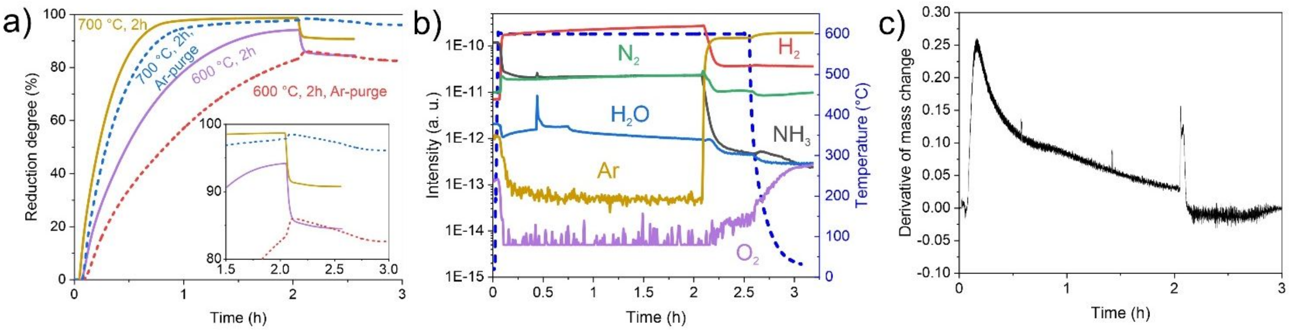

Supplementary Figure S7: a) Comparison of the reduction degree curves of the ADR samples reduced at 600 and 700 ˚C for 2 h and subsequently cooled in ammonia gas (solid curves), with the ADR samples reduced at same temperatures and reduction time with the cooling performed in Ar gas (dotted curves). After 2 h reduction time and before the cooling procedure, purging was performed with Ar gas for 30 minutes. The insert in a) provides a detailed view into the modified change of the weight of the sample with different cooling and reduction temperatures. b) Exemplary gas evolution obtained from in-situ measurements with a mass spectrometer for the ADR sample reduced at 600 ˚C for 2 h with subsequent cooling in Ar. c) Corresponding derivative of the mass change of the ADR sample reduced at 600 ˚C for 2 h with subsequent cooling in Ar. The corresponding difference in the reduction progression and degree between samples 600 ˚C 2 h and 600 ˚C 2 h with Ar-purging is related to the varying heterogeneity between samples. Please see Text S3 for more details.

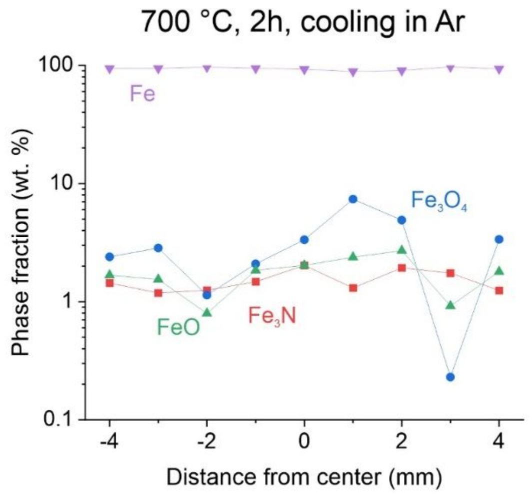

Supplementary Figure S8: Cross-sectional phase composition of ADR pellet samples reduced at 700 ˚C for 2 h with cooling performed in Ar gas.

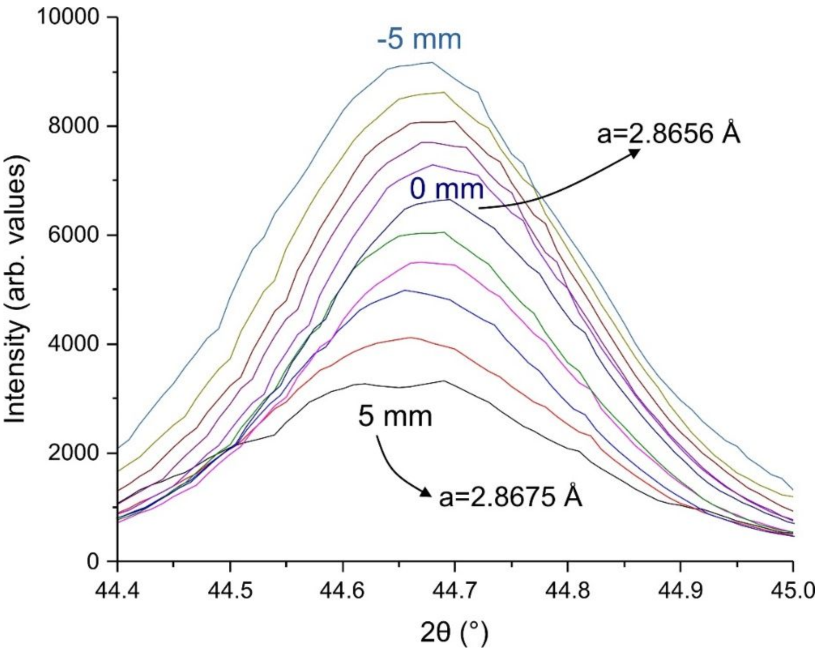

Supplementary Figure S9: Enlarged portion of the XRD diffractograms at main {101} iron peak of the cross-section of ADR sample reduced at 600 ˚C for 2 h and cooled in Ar gas. The

measurements are done with 1 mm steps and the diffractograms are stacked in a waterfall form from one edge to the other of the cross-section. The determined lattice parameter for the 0 mm position and maximum shift from the edge position (5 mm) is denoted in the image.

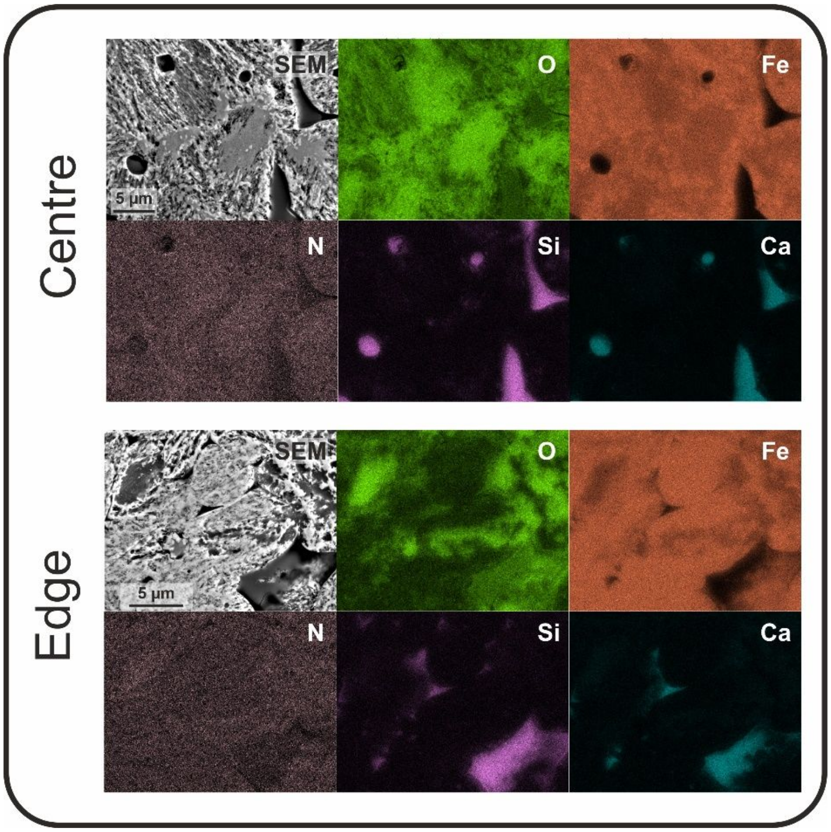

Supplementary Figure S10: Secondary electron images (SEI) and elemental distribution maps obtained with electron dispersion X-ray spectroscopy (EDX) for ADR pellet reduced at 600 ˚C for 2 h with cooling in Ar and after 4 days exposure in air. The different regions represent a typical microstructure present in the different portions of the pellet.

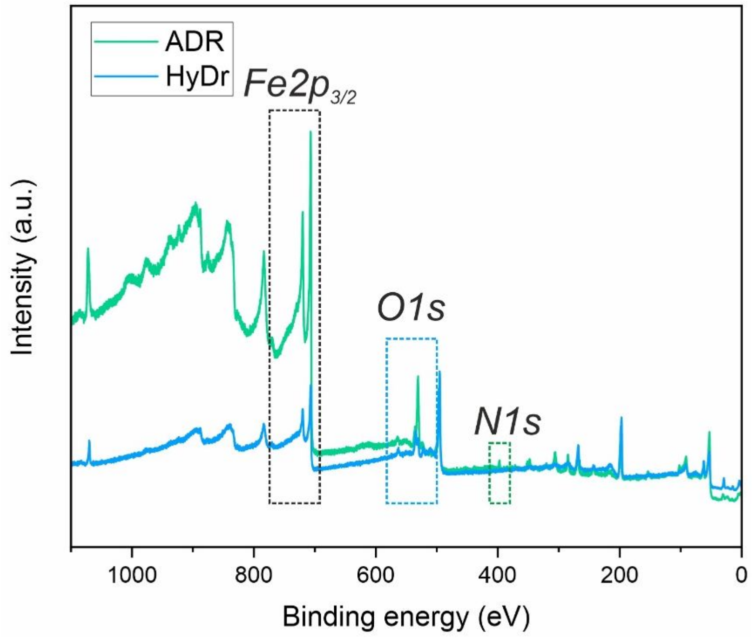

Supplementary Figure S11: Overview XPS spectra of both ADR and HyDR samples. The individual marked regions correspond to the regions of interested that were investigated with high-resolution XPS scans.

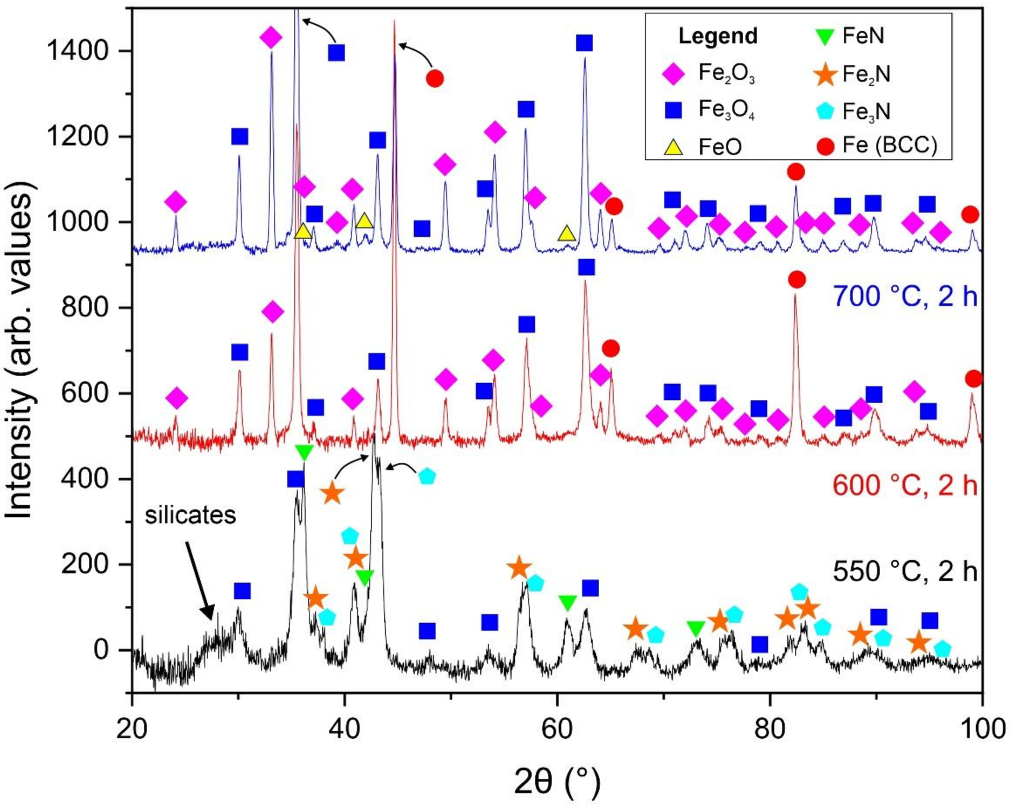

Supplementary Figure S42: XRD measurements of the surface of the reduced pellets of iron oxide, reduced with ADR at 550 ˚C, 600 ˚C and 700 ˚C for 2 h. The cooling of the pellets was performed in Ar gas. The corresponding major phases are marked with designated symbols. For the sake of clarity some of the minor peaks are not marked.

REFERENCES

(1) Vogl, V.; Åhman, M.; Nilsson, L. J. Assessment of Hydrogen Direct Reduction for Fossil-Free Steelmaking. *J Clean Prod* **2018**, *203*, 736–745. https://doi.org/10.1016/J.JCLEPRO.2018.08.279.

(2) Geng, J.; Sun, H. Optimization and Analysis of a Hydrogen Liquefaction Process: Energy, Exergy, Economic, and Uncertainty Quantification Analysis. *Energy* **2023**, *262*, 125410. https://doi.org/10.1016/J.ENERGY.2022.125410.

(3) *Liquid hydrogen transport | Cryolor*. https://www.cryolor.com/cryogenic-transport/liquid-hydrogen-transport (accessed 2024-01-25).

(4) Gautam, M.; Rao, K. V. S.; Saxena, B. K. Reduction in Liquid Hydrogen by Weight Due to Storage in Different Sizes of Containers for Varying Period of Time. *Proceedings of 2017 IEEE International Conference on Technological Advancements in Power and Energy: Exploring Energy Solutions for an Intelligent Power Grid, TAP Energy 2017* **2018**, 1–6. https://doi.org/10.1109/TAPENERGY.2017.8397347.

(5) Pissot, S.; Thunman, H.; Samuelsson, P.; Seemann, M. Production of Negative-Emissions Steel Using a Reducing Gas Derived from DFB Gasification. *Energies 2021, Vol. 14, Page 4835* **2021**, *14* (16), 4835. https://doi.org/10.3390/EN14164835.

(6) Katebah, M.; Al-Rawashdeh, M.; Linke, P. Analysis of Hydrogen Production Costs in Steam-Methane Reforming Considering Integration with Electrolysis and CO2 Capture. *Clean Eng Technol* **2022**, *10*, 100552. https://doi.org/10.1016/J.CLET.2022.100552.

(7) Patisson, F.; Mirgaux, O. Hydrogen Ironmaking: How It Works. *Metals 2020, Vol. 10, Page 922* **2020**, *10* (7), 922. https://doi.org/10.3390/MET10070922.

(8) Bhaskar, A.; Assadi, M.; Somehsaraei, H. N. Can Methane Pyrolysis Based Hydrogen Production Lead to the Decarbonisation of Iron and Steel Industry? *Energy Conversion and Management: X* **2021**, *10*, 100079. https://doi.org/10.1016/J.ECMX.2021.100079.

(9) Ma, Y.; Bae, J. W.; Kim, S.-H.; Jovičević-Klug, M.; Li, K.; Vogel, D.; Ponge, D.; Rohwerder, M.; Gault, B.; Raabe, D. Reducing Iron Oxide with Ammonia: A Sustainable Path to Green Steel. **2023**. https://doi.org/10.1002/advs.202300111.

(10) Kojima, Y. Safety of Ammonia as a Hydrogen Energy Carrier. *Int J Hydrogen Energy* **2024**, *50*, 732–739. https://doi.org/10.1016/J.IJHYDENE.2023.06.213.

(11) Klerke, A.; Christensen, C. H.; Nørskov, J. K.; Vegge, T. Ammonia for Hydrogen Storage: Challenges and Opportunities. *J Mater Chem* **2008**, *18* (20), 2304–2310. https://doi.org/10.1039/B720020J.

(12) Song, Q.; Tinoco, R. R.; Yang, H.; Yang, Q.; Jiang, H.; Chen, Y.; Chen, H. A Comparative Study on Energy Efficiency of the Maritime Supply Chains for Liquefied Hydrogen, Ammonia, Methanol and Natural Gas. *Carbon Capture Science & Technology* **2022**, *4*, 100056. https://doi.org/10.1016/J.CCST.2022.100056.

(13) Andersson, J.; Krüger, A.; Grönkvist, S. Methanol as a Carrier of Hydrogen and Carbon in Fossil-Free Production of Direct Reduced Iron. *Energy Conversion and Management: X* **2020**, *7*, 100051. https://doi.org/10.1016/J.ECMX.2020.100051.

(14) Reddy, V. J.; Hariram, N. P.; Maity, R.; Ghazali, M. F.; Kumarasamy, S. Sustainable E-Fuels: Green Hydrogen, Methanol and Ammonia for Carbon-Neutral Transportation. *World Electric Vehicle Journal 2023, Vol. 14, Page 349* **2023**, *14* (12), 349. https://doi.org/10.3390/WEVJ14120349.

(15) Spatolisano, E.; Pellegrini, L. A.; de Angelis, A. R.; Cattaneo, S.; Roccaro, E. Ammonia as a Carbon-Free Energy Carrier: NH3 Cracking to H2. *Ind Eng Chem Res* **2023**, *62* (28), 10813–10827. https://doi.org/10.1021/ACS.IECR.3C01419/ASSET/IMAGES/LARGE/IE3C01419_0005.JPEG.

(16) Gubbi, S.; Cole, R.; Emerson, B.; Noble, D.; Steele, R.; Sun, W.; Lieuwen, T. Air Quality Implications of Using Ammonia as a Renewable Fuel: How Low Can NOx Emissions Go? *ACS Energy Lett* **2023**, *8* (10), 4421–4426. https://doi.org/10.1021/ACSENERGYLETT.3C01256/ASSET/IMAGES/LARGE/NZ3C01256_0006.JPEG.

(17) Elbaz, A. M.; Wang, S.; Guiberti, T. F.; Roberts, W. L. Review on the Recent Advances on Ammonia Combustion from the Fundamentals to the Applications. *Fuel Communications* **2022**, *10*, 100053. https://doi.org/10.1016/J.JFUECO.2022.100053.

(18) Chai, W. S.; Bao, Y.; Jin, P.; Tang, G.; Zhou, L. A Review on Ammonia, Ammonia-Hydrogen and Ammonia-Methane Fuels. *Renewable and Sustainable Energy Reviews* **2021**, *147*, 111254. https://doi.org/10.1016/J.RSER.2021.111254.

(19) Shen, C. Y.; Liou, S. Y. Surface Acoustic Wave Gas Monitor for Ppm Ammonia Detection. *Sens Actuators B Chem* **2008**, *131* (2), 673–679. https://doi.org/10.1016/J.SNB.2007.12.061.

(20) Smyntyna, V.; Consales, M.; Viter, R.; Pisco, M.; Campopiano, S.; Cusano, A.; Giordano, M. A Novel Optochemical Sensor Based on SnO_2 Sensitive Thin Film for Ppm Ammonia Detection in Liquid Environment. *Journal of Lightwave Technology, Vol. 24, Issue 12, pp. 5000-5007* **2006**, *24* (12), 5000–5007.

(21) Smeets, M. A. M.; Bulsing, P. J.; van Rooden, S.; Steinmann, R.; de Ru, J. A.; Ogink, N. W. M.; van Thriel, C.; Dalton, P. H. Odor and Irritation Thresholds for Ammonia: A Comparison between Static and Dynamic Olfactometry. *Chem Senses* **2007**, *32* (1), 11–20. https://doi.org/10.1093/CHEMSE/BJL031.

(22) Jeong, S. Y.; Jang, D.; Lee, M. C. Property-Based Quantitative Risk Assessment of Hydrogen, Ammonia, Methane, and Propane Considering Explosion, Combustion, Toxicity, and Environmental Impacts. *J Energy Storage* **2022**, *54*, 105344. https://doi.org/10.1016/J.EST.2022.105344.

(23) Spreitzer, D.; Schenk, J. Reduction of Iron Oxides with Hydrogen—A Review. *Steel Res Int* **2019**, *90* (10), 1900108. https://doi.org/10.1002/SRIN.201900108.

(24) Kiełbasa, K.; Pelka, R.; Arabczyk, W. Studies of the Kinetics of Ammonia Decomposition on Promoted Nanocrystalline Iron Using Gas Phases of Different Nitriding Degree. *Journal of Physical Chemistry A* **2010**, *114* (13), 4531–4534. https://doi.org/10.1021/JP9099286.

(25) Yeo, S. C.; Han, S. S.; Lee, H. M. Mechanistic Investigation of the Catalytic Decomposition of Ammonia (NH3) on an Fe(100) Surface: A DFT Study. *Journal of Physical Chemistry C* **2014**, *118* (10), 5309–5316. https://doi.org/10.1021/JP410947D.

(26) Ko, Y.; Ima,]; Inouye, M.; Yamada, Y. Solubility and Diffusivity of Nitrogen in Liquid Iron-Nickel and Iron-Cobalt Alloys at 1600°C*.

(27) Simmons, J. W. Overview: High-Nitrogen Alloying of Stainless Steels. *Materials Science and Engineering: A* **1996**, *207* (2), 159–169. https://doi.org/10.1016/0921-5093(95)09991-3.

(28) Pitkälä, J.; Holappa, L.; Jokilaakso, A. A Study of the Effect of Alloying Elements and Temperature on Nitrogen Solubility in Industrial Stainless Steelmaking. *Metallurgical and Materials Transactions B: Process Metallurgy and Materials Processing Science* **2022**, *53* (4), 2364–2376. https://doi.org/10.1007/S11663-022-02534-1/FIGURES/7.

(29) Dellacorte, C. Gas Nitriding. *Encyclopedia of Tribology* **2013**, 1455–1460. https://doi.org/10.1007/978-0-387-92897-5_719.

(30) Aufrecht, J.; Leineweber, A.; Mittemeijer, E. J.; Foct, J. The Structure of Nitrogen-Supersaturated Ferrite Produced by Ball Milling. *Philosophical Magazine* **2008**, *88* (12), 1835–1855. https://doi.org/10.1080/14786430802322198ï.

(31) El-Zoka, A. A.; Stephenson, L. T.; Kim, S. H.; Gault, B.; Raabe, D. The Fate of Water in Hydrogen-Based Iron Oxide Reduction. *Advanced Science* **2023**, *10* (24), 2300626. https://doi.org/10.1002/ADVS.202300626.

(32) Kim, S. H.; Zhang, X.; Ma, Y.; Souza Filho, I. R.; Schweinar, K.; Angenendt, K.; Vogel, D.; Stephenson, L. T.; El-Zoka, A. A.; Mianroodi, J. R.; Rohwerder, M.; Gault, B.; Raabe, D. Influence of Microstructure and Atomic-Scale Chemistry on the Direct Reduction of Iron Ore with Hydrogen at 700°C. *Acta Mater* **2021**, *212*, 116933. https://doi.org/10.1016/J.ACTAMAT.2021.116933.

(33) Bai, Y.; Mianroodi, J. R.; Ma, Y.; da Silva, A. K.; Svendsen, B.; Raabe, D. Chemo-Mechanical Phase-Field Modeling of Iron Oxide Reduction with Hydrogen. *Acta Mater* **2022**, *231*, 117899. https://doi.org/10.1016/J.ACTAMAT.2022.117899.

(34) Ma, Y.; Souza Filho, I. R.; Zhang, X.; Nandy, S.; Barriobero-Vila, P.; Requena, G.; Vogel, D.; Rohwerder, M.; Ponge, D.; Springer, H.; Raabe, D. Hydrogen-Based Direct Reduction of Iron Oxide at 700°C: Heterogeneity at Pellet and Microstructure Scales. *International Journal of Minerals, Metallurgy and Materials* **2022**, *29* (10), 1901–1907. https://doi.org/10.1007/S12613-022-2440-5/METRICS.

(35) Idriss, H. On the Wrong Assignment of the XPS O1s Signal at 531–532 EV Attributed to Oxygen Vacancies in Photo- and Electro-Catalysts for Water Splitting and Other Materials Applications. *Surf Sci* **2021**, *712*, 121894. https://doi.org/10.1016/J.SUSC.2021.121894.

(36) Dhankhar, S.; Bhalerao, G.; Ganesamoorthy, S.; Baskar, K.; Singh, S. Growth and Comparison of Single Crystals and Polycrystalline Brownmillerite Ca2Fe2O5. *J Cryst Growth* **2017**, *468*, 311–315. https://doi.org/10.1016/J.JCRYSGRO.2016.09.051.

(37) Cai, Y.; Pan, Y.; Xue, J.; Sun, Q.; Su, G.; Li, X. Comparative XPS Study between Experimentally and Naturally Weathered Pyrites. *Appl Surf Sci* **2009**, *255* (21), 8750–8760. https://doi.org/10.1016/J.APSUSC.2009.06.028.

(38) Tian, Z.; Wang, C.; Si, Z.; Wen, C.; Xu, Y.; Lv, W.; Chen, L.; Zhang, X.; Ma, L. Enhancement of Light Olefins Selectivity Over N-Doped Fischer-Tropsch Synthesis Catalyst Supported on Activated Carbon Pretreated with KMnO4. *Catalysts 2019, Vol. 9, Page 505* **2019**, *9* (6), 505. https://doi.org/10.3390/CATAL9060505.

(39) Emrich, R. J.; Traynor, L.; Gambogi, W.; Buhks, E. Surface Analysis of Electrochromic Displays of Iron Hexacyanoferrate Films by X-ray Photoelectron Spectroscopy. *Journal of Vacuum Science & Technology A* **1987**, *5* (4), 1307–1310. https://doi.org/10.1116/1.574797.
